# Explaining graph convolutional network predictions for clinicians—An explainable AI approach to Alzheimer's disease classification

**DOI:** 10.3389/frai.2023.1334613

**Published:** 2024-01-08

**Authors:** Sule Tekkesinoglu, Sara Pudas

**Affiliations:** ^1^Department of Computing Science, Umeå University, Umeå, Sweden; ^2^Department of Integrative Medical Biology (IMB), Umeå University, Umeå, Sweden; ^3^Umeå Center for Functional Brain Imaging, Umeå University, Umeå, Sweden

**Keywords:** explainable AI, multimodal data, graph convolutional networks, Alzheimer's disease, node classification

## Abstract

**Introduction:**

Graph-based representations are becoming more common in the medical domain, where each node defines a patient, and the edges signify associations between patients, relating individuals with disease and symptoms in a node classification task. In this study, a Graph Convolutional Networks (GCN) model was utilized to capture differences in neurocognitive, genetic, and brain atrophy patterns that can predict cognitive status, ranging from Normal Cognition (NC) to Mild Cognitive Impairment (MCI) and Alzheimer's Disease (AD), on the Alzheimer's Disease Neuroimaging Initiative (ADNI) database. Elucidating model predictions is vital in medical applications to promote clinical adoption and establish physician trust. Therefore, we introduce a decomposition-based explanation method for individual patient classification.

**Methods:**

Our method involves analyzing the output variations resulting from decomposing input values, which allows us to determine the degree of impact on the prediction. Through this process, we gain insight into how each feature from various modalities, both at the individual and group levels, contributes to the diagnostic result. Given that graph data contains critical information in edges, we studied relational data by silencing all the edges of a particular class, thereby obtaining explanations at the neighborhood level.

**Results:**

Our functional evaluation showed that the explanations remain stable with minor changes in input values, specifically for edge weights exceeding 0.80. Additionally, our comparative analysis against SHAP values yielded comparable results with significantly reduced computational time. To further validate the model's explanations, we conducted a survey study with 11 domain experts. The majority (71%) of the responses confirmed the correctness of the explanations, with a rating of above six on a 10-point scale for the understandability of the explanations.

**Discussion:**

Strategies to overcome perceived limitations, such as the GCN's overreliance on demographic information, were discussed to facilitate future adoption into clinical practice and gain clinicians' trust as a diagnostic decision support system.

## 1 Introduction

Real-world data often comes in multiple modalities, such as image, text, and numerical data. These data are often relational, for example, patient medical records where various modalities contribute to a single outcome in the medical field. Humans are experts at multimodal thinking, able to effortlessly incorporate new inputs into a knowledge space shaped by experience. However, AI systems in the medical domain face a complex challenge in the integration, fusion, and mapping of various distributed and heterogeneous data in diagnostic modeling. It is essential to consider that different features from diverse data types contribute to an outcome. Graphs provide a way of information fusion for multimodal data and enable an intuitive way of modeling patients and associations between them (Holzinger et al., 2021).

Parallel to the previous research, our study involves working with multimodal data, which we modeled as a graph. Each node in the graph represents a set of patient data, and the pairwise correlations between nodes are represented as edges. Similar patients are embedded close to each other in an edge-weighted graph approximating similarity in the network. This also reveals the relationship between integrated modalities and disease. Unlike previous studies (Parisot et al., 2018; Anirudh and Thiagarajan, 2019), which only looked at neuroimaging data, our study includes genetic, cognitive, and neuropsychological test results in the nodal features. Additionally, the functional connectivity matrices provide information to establish the association between the patients' feature vectors.

**Figure 1:**
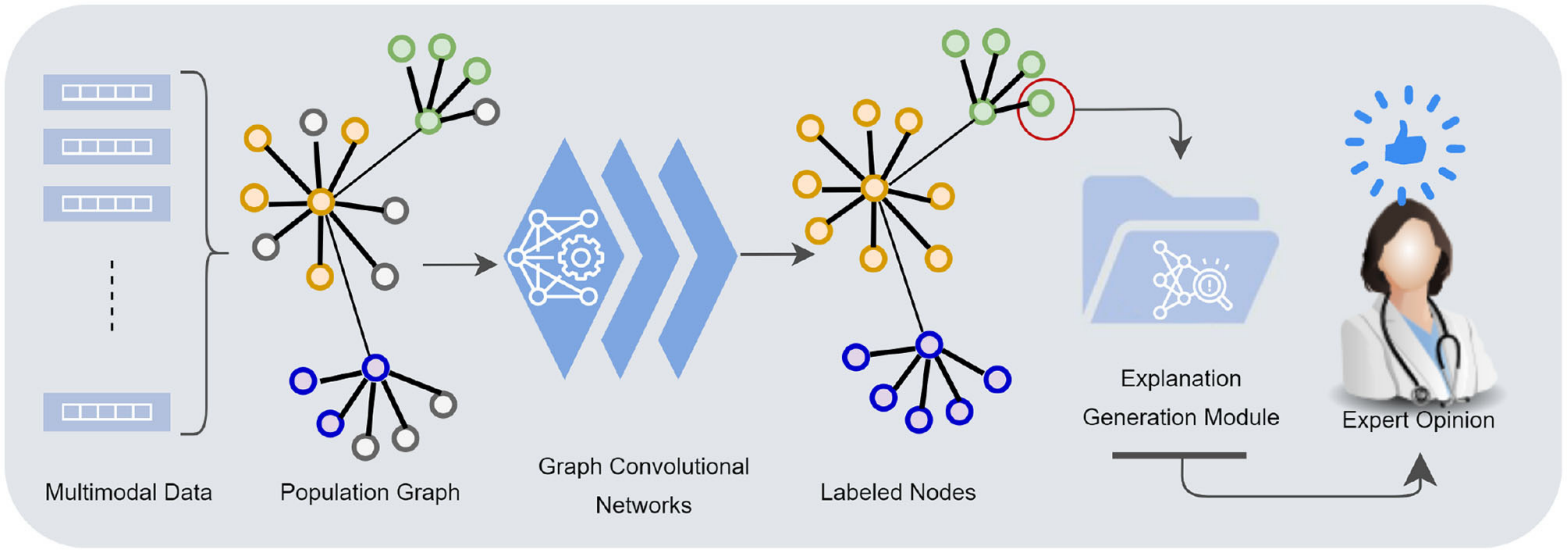
Multimodal data—demographics, imaging, neurocognitive tests, biomarkers—are mapped into a population graph. Graph convolutional networks learn from multimodal data taking the similarity structures of the graph into account. The explanation generation module presents the model's underlying rationale for individual node classification to a human counterpart.

Although GCNs are highly effective at modeling a wide range of complex and diverse data, they inherently lack transparency. While accuracy is a critical factor in establishing trust, interpretability is equally crucial for integrating these models into clinical applications. Medical professionals are understandably hesitant to rely solely on machine learning predictions and require evidence and interpretation, especially in diagnosing disease. Our work introduces a decomposition-based explanation method for individual node classification, allowing for a clear understanding of how various features from different modalities contribute to a diagnostic result for a specific patient (see Figure 1).

Taken together, we investigate the potential utility of a GCN model for multimodal data in a node classification task and provide justifications for predictions made by the model. Furthermore, we conducted a human subject study with domain experts to assess whether it allows for a meaningful explanation in a way that physicians can understand. The main contributions of the proposed work are:

- An extended semi-supervised GCN model for population graph, integrating multimodal data in multiclass classification problem.
- Introducing decomposition-based explanation method for individual node, i.e., patient, classification.
- Objective and human-grounded evaluations to assess stability, correctness, and comprehensibility of the explanation.

The rest of the paper is organized as follows: Section 2 discusses the relevant work. Section 3 presents the methodology, describing the feature selection, data preparation, the graph model, and the explanation method. Section 4 provides details of the evaluation method and results. The findings are discussed in Section 5, and Section 6 concludes the paper.

## 2 Background

Multimodal data analysis is increasing in prevalence due to the limitations of single modalities, resulting in increasingly complex data sets. In recent years, there has been an increasing trend in works proposing a combination of multiple modalities for Alzehimer disease-related tasks, including diagnosis, conversion prediction, and progression detection. For disease diagnosis tasks, many proposed Random Forest and XGBoost models (El-Sappagh et al., 2021; Bogdanovic et al., 2022; Lombardi et al., 2022), and others proposed multimodal learning-based approaches using deep learning to summarize features of all modalities (Kamal et al., 2021; Ilias and Askounis, 2022; Mulyadi et al., 2022; Rahim et al., 2022) (see Table 1 for details). Previous research has generally overlooked the interactions and associations between subjects in a population. modeling similarities between subjects can improve learned representations of multimodal data and classification performance. Graphs provide a natural way to represent the population data and model complex interactions by combining features of different modalities for disease analysis (Parisot et al., 2017; Anirudh and Thiagarajan, 2019; Yao et al., 2019; Rakhimberdina et al., 2020). The association among instances is critical where information from neighboring patients in the graph provides information about a subject's status.

**Table 1:** Recent works on multimodal data use in AD disease diagnosis-related tasks proposing explainable machine learning approaches.

**Reference**	**Modalities**	**ML method**	**Task**	**Explainability method**	**Evaluation**	
					**Model**	**Explanation**
Rahim et al. (2022)	MRI, demographic, cognitive test results	3D-CNN BRNN	AD progression detection	Time-series grad cam	ACC., PRE., SEN., AUC	Literature comparison
El-Sappagh et al. (2021)	MRI, demographic, genetic, cognitive, neuropsychology. test results	Random Forest	AD diagnosis and progression	Shapley values derivative	ACC. and F1	Case study and comparison with shapley values
Lombardi et al. (2022)	Demographic, clinical, neuropsychology. test results	Random Forest	AD diagnosis	Shapley values	ACC., PRE., SEN., AUC	Longitudinal analysis (stability)
Bogdanovic et al. (2022)	Medical history, cognitive test results, and lifestyle	XGBoost	AD diagnosis	Shapley values	ACC., PRE., recall, SPE., F1	None
Mulyadi et al. (2022)	MRI, demographic, cognitive test results, and stages	Deep Learning	AD progression	Likelihood map estimation	ACC., AUC, F1	None
Kamal et al. (2021)	MRI and gene expression	SpinalNet CNN	Dementia classification (Mild, moderate, very moderate)	LIME	ACC., pre., recall, F1	None
Ilias and Askounis (2022)	Demographic, cognitive test results, and text (linguistic responses)	MTL BERT	Dementia classification (Mild, moderate, very moderate)	LIME	ACC., PRE., recall, SPEC, F1	Self assessment
Danso et al. (2021)	Demographic, medical history, lifestyle	Random forest and XGBoost	Dementia risk prediction	Shapley values	ACC., AUC, SEN, SPEC, p-value	Self assessment
Velazquez et al. (2021)	Demographic, brain volume, and cognitive test results	Random Forest	Conversion prediction (MCI to AD)	Feature importance	ACC., PRE., recall, F1, AUC, p value	Ablation study
Ours	Demographic, MRI, biomarker, cognitive, neuropsychology. test results	GCN	AD classification (NC, MCI, AD)	Decomposition based feature importance	ACC., PRE., recall, F1	User study, comparison with SHAP, objective evaluation (stability)

ACC., Accuracy; AUC, Area under the curve; PRE., Precision; SEN., Sensitivity; SPEC., Specificity.

Researchers have recently employed graph convolutional networks to study their advantage in population graphs. Each subject is modeled as a node along with a set of features, and the graph edges are defined based on the similarity between the features of the subjects (Rakhimberdina et al., 2020). They have been shown to be effective, specifically for brain disorder classification. Parisot et al. (2017, 2018) proposed a spectral GCN model which takes into account both the pairwise similarity between subjects (phenotypic information) and information obtained from subject-specific imaging features to classify individuals as healthy control or patient (i.e., Autism Spectrum Disorder and Alzheimer's Disease) in a population. Rakhimberdina and Murata (2019) applied a linear simple graph convolution (SGC) (Wu et al., 2019) using the distance between phenotypic features of the subjects as weights of the edges of the graph. In our work, we follow the similar efficient practice proposed by previous works and advance it by fusing features from cognitive, neuropsychological, and biomarker data, which are highly relevant and critical to diagnosing Alzheimer's disease.

### 2.1 Explanation methods

Machine learning models have shown great potential in diagnosing Alzheimer's disease (AD), but the lack of explainability poses a challenge for their clinical application (Zhang et al., 2022). Researchers have been exploring explainability approaches to address the black-box nature of these methods. There are two types of Explainable AI (XAI) approaches that have been adapted into clinical applications: *intrinsic* and *post-hoc*. Intrinsic methods allow us to understand the decision-making process or the basis of a technique without external interpretation methods. In contrast, post-hoc methods aim to retrospectively understand which part of the input data accounts for the classification decision for any classifier (Ghanvatkar and Rajan, 2022). Post-hoc explanation methods including SHapley Additive exPlanations (SHAP) (Danso et al., 2021; El-Sappagh et al., 2021; Lombardi et al., 2022), Local Interpretable Model-agnostic Explanations (LIME) (Kamal et al., 2021; Ilias and Askounis, 2022), time series grad-cam (Rahim et al., 2022), feature importance (Velazquez et al., 2021), and likelihood map estimation (Mulyadi et al., 2022) have been adapted into models for AD diagnosis-related tasks. Interpretability for graph-based deep learning is even more challenging than CNN or Recurrent Neural Network (RNN) based models since graph nodes and edges are often heavily interconnected. Model-specific and model-agnostic post-hoc interpretability are the two most common approaches for GCNs. The former explainability approaches are designed exclusively for specific graph models, constraining other models from providing details about the uncovered relationships (such as sparsity and modularity) (Ahmedt-Aristizabal et al., 2021). The latter increases the generalizability of the explanation method in applying to various graph-based learning algorithms. Several XAI methods have been redesigned and applied to GNNs, including layer-wise relevance propagation (LRP) (Baldassarre and Azizpour, 2019; Schwarzenberg et al., 2019), excitation backpropagation (Pope et al., 2019), graph pruning (Ying et al., 2019), and gradient-based saliency (Chattopadhay et al., 2018).

The prior studies, specifically feature importance-based explanation methods, generally focus on the impact of a feature at the individual level and overlook the influence of features at the group level, such as a whole modality. In our approach to explanation, we incorporate relational information by focusing on both the node features (individual and group) and edges, estimating the impact of different patient groups (e.g., normal control, mild cognitive) using edge weights. Another area for improvement is the presentation of the explanations. It is important to tailor the design of explanations to the intended goals and user groups, especially in clinical settings. Clinicians have unique characteristics and needs that differ from general users, and as such, explanations should be constructed using domain-specific concepts and an appropriate level of detail that corresponds to their background. Our work focuses on providing interpretability that is specifically designed for physicians in clinical contexts. We present explanations in a visual format with textual descriptions to support comprehension.

### 2.2 Evaluation of the explanation methods

Medical AI research aims to build applications that use AI technologies to assist doctors in making medical decisions. Explanations for predictions made by ML models are necessary for making high-stakes clinical decisions which can aid by providing relevant and domain-aware information. While many XAI techniques have been designed to generate explanations for black-box model predictions, there are no rigorous metrics to evaluate these explanations concerning their consistency and comprehensibility to clinicians (Ghanvatkar and Rajan, 2022). In general, there need to be common ground evaluation strategies for assessing the usefulness of explanations in medical applications.

The proposal of different explanation methods compelled researchers to introduce various evaluation metrics to assess how well the model fits in a certain aspect of explainability. Generally, there are two main ways to evaluate explanation methods: objective evaluations and human-grounded evaluations. Objective evaluation includes objective metrics and automated approaches to assessing the functional properties of the explanation methods (e.g., fidelity, stability, explanatory power) (Doshi-Velez and Kim, 2017). Human-grounded evaluation develops methods with a human-in-the-loop approach by utilizing end-users feedback and their informed opinion, whether with experts or laypersons (novice users) (Vilone and Longo, 2020). Human-centered studies with domain experts are especially important for clinical applications to gain informed judgment on the explanations produced by the model and verify the consistency of the explanations with the domain knowledge. Studies involving highly-trained domain experts are more challenging due to the difficulty of accessing and compensating for it, which therefore is omitted by many studies.

While many works evaluate the model's performance and accuracy, there is a lack of both objective and clinical assessment of the explanation module. Some of the studies presented case studies (El-Sappagh et al., 2021), literature comparison (Rahim et al., 2022), and assessing the stability of the explanations through longitudinal analysis (Lombardi et al., 2022). In contrast to previous work, we evaluate our results with experts in AD diagnostics to assess the human agreement on the model's prediction and rationale and the quality of the explanations provided by the model.

## 3 Materials and methods

Our methodology consists of multiple steps, commencing with data preparation and constructing graphs, followed by training a graph convolutional network and generating post-hoc explanations for node classification. We aim to incorporate the most relevant clinical and imaging data in the ADNI database to train the predictive model and explain how each feature contributes to a predicted outcome. The techniques proposed in this work are evaluated experimentally by measuring the property of individual explanations and conducting a human-subject study with experts on AD diagnostics. The following subsections describe the methodology in detail.

### 3.1 Feature selection and data preparation

Diagnosing Alzheimer's disease benefits from thorough medical evaluation with several tests and procedures, including an MRI scan of the brain, neuropsychology tests of memory and thinking, and positron emission tomography (PET) to identify certain biomarkers. The Alzheimer's Disease Neuroimaging Initiative (ADNI)^1^ provides an extensive repository of clinical and neuroimaging data to advance the understanding AD pathophysiology and improve diagnostic methods for early AD detection (Jack et al., 2008). The ADNI study has been tracking the progression of the disease using biomarkers, together with clinical measures, to assess the brain's structure and function for four disease states (AD, MCI, Late-MCI, Early-MCI). The study enrolls participants between the ages of 55 and 90 at 57 sites in the United States and Canada in four phases (ADNI GO, 1, 2, 3). During these phases, participants are carried forward from previous phases for continued monitoring, while new participants are added with each phase to investigate the evolution of Alzheimer's disease further. In this work, we have gathered the phenotypic, clinical, and imaging data under five categories for participants from all four phases (i.e., demographics, cognitive tests, neuropsychology tests, medical imaging, and biomarker/genetic data). We aim to include the most important aspects of AD diagnosis, where various modalities contribute to a single outcome.

Demographics include the patient's age and gender information. Although these attributes are not directly related to detecting or classifying Alzheimer's disease, they are the main risk factors for developing AD (Vina and Lloret, 2010). Gender differences have been observed in several studies suggesting that the risk of developing certain subtypes of dementia differs (i.e., AD vs vascular dementia) between females and males (Podcasy and Epperson, 2022). Similarly, studies on brain structures of typical aging and early Alzheimer's disease pathology demonstrate varying atrophy patterns (Raji et al., 2009).

Cognitive test results include the scores related to memory (MEM), executive function (EXF), and language (LAN). These values are in composite form as these domains are measured to a different extent in the ADNI study. Memory is one of the domains which is measured quite extensively. Earlier works show that memory and executive function are highly predictive of disease progression (Giorgio et al., 2020). Other neuropsychological test includes Geriatric Depression Scale (GDS), Montreal Cognitive Assessment (MoCA), and Mini-Mental State Examination score (MMSE). GDS is an affective measure which shown to be predictive of MCI conversion to AD in moderate to severe depressive symptoms (i.e., GDS > 15) (Defrancesco et al., 2017). MoCA and MMSE are other neuropsychological tests that are designed to measure psychological functions (i.e., sensing, feeling, thinking, intuition) linked to a particular brain structure (Boyle et al., 2012). We note that MoCA and MMSE could be described as both neuropsychological and cognitive tests.

Alzheimer's is characterized by predominant damage to the brain's temporal lobe, and the extent of damage often extends to other areas (Hill, 2022). Atrophy patterns of typical aging and early Alzheimer's disease pathology studied on brain structures show atrophy mainly in the anterior hippocampal/parahippocampal regions and the precuneus in AD patients (Raji et al., 2009). Hence, we have selected the mean cortical thickness of the temporal lobe (including entorhinal, fusiform, superior temporal, inferior temporal, middle temporal, parahippocampal, banks of the superior temporal sulcus, temporal pole regions) combined with mean precuneus cortical thickness and total hippocampus volume. The data is derived from volumetric segmentation of longitudinal T1 MR images through FreeSurfer (Fischl, 2012; Reuter et al., 2012) (see Supplementary Figure S1 for visualization of the selected regions).

Biomarkers data includes β-Amyloid (Aβ), tauopathy, and Polygenic Hazard Score (PHS). Positivity for β-Amyloid (Aβ) was based on positron emission tomography imaging, while tau positivity was based on P-tau 181 measured in plasma, according to standard cut-off values as provided in the ADNI dataset. PHS is generated based on the combination of APOE and 31 other genetic variants (Desikan et al., 2017). These are the hallmark pathologies of AD, which in fact, appear before cognitive problems show up. It helps identify asymptomatic individuals potentially at risk for a future clinical disorder.

Data from the selected features are merged based on the participants' ID and visit code. In total, 2,212 samples were available, with 363 individuals diagnosed with Alzheimer's Disease (AD), 1095 mild cognitive impairment (MCI), and 754 Normal Controls (NC). The categories are based on the clinical diagnosis of the patients given along with the cognitive test scores. We also note that some patients have multiple data points in the resulting data set.

The feature values outside the lower and upper percentile threshold (outliers) (0.05–0.95) and missing values are replaced with a moving mean using a window length of 8 within the class. Statistical analysis of the dataset indicates that samples significantly differ from one another in different groups (*p* = 0 | p < 0.05) (see Table 2). As expected, AD has more differentiating feature values (MEM = -0.84 ± 0.39, LAN = -0.24 ± 0.47, EXF = -0.41 ± 0.63) than the other two classes, which is an advantage considering the sample size of this group. MCI had overlapping features with the NC group to some extent concerning neuropsychological (MMSE 29.1 ± 1.07 to 28.1 ± 1.76) and imaging features (MCT 2.84 ± 0.10 to 2.78 ± 0.15, THV 6.54 ± 0.56 to 6.34 ± 0.70).

**Table 2:** Feature analysis on the dataset for NC, MCI, and AD node classification task.

		**NC**	**MCI**	**AD**	*P*-value*

Sample size		754	1,095	363	
Demographic	Age	75.6. ± 6.7	**73.7** **±7.5**	75.1 ± 7.9	1.5215 × 10-7
	GEN (F/M)	**399/355**	458/637	160/203	2.5176 × 10-58
Cognitive	MEM	0.93 ± 0.41	0.33 ± 0.50	**-0.84** **±0.39**	0
	LAN	0.88 ± 0.36	0.56 ± 0.38	**-0.24** **±0.47**	1.0955 × 10-307
	EXF	0.85 ± 0.41	0.53 ± 0.42	**-0.41** **±0.63**	7.2301 × 10-291
Neuropsychological	GDS	**7.1** **±1.34**	8.3 ± 2.09	9.2 ± 2.63	7.8077 × 10-66
	MoCA	26.3 ± 2.57	23.2 ± 3.36	**14.8** **±3.48**	0
	MMSE	29.1 ± 1.07	28.1 ± 1.76	**22.4** **±3.32**	0
Medical imaging	MCT(*mm*)	2.84 ± 0.10	2.78 ± 0.15	**2.49** **±0.19**	1.9123 × 10-252
	THV(*cm*^3^)	6.54 ± 0.56	6.34 ± 0.70	**5.42** **±0.72**	3.5133 × 10-137
Biomarker/genetic	Aβ±	441/313	493/602	**31/332**	2.5176 × 10-58
	Tau±	312/442	344/751	**7/356**	5.4999 × 10-314
	PHS	0.007 ± 0.49	0.29 ± 0.65	**0.84** **±0.64**	1.4324 × 10-93

Data is shown as mean ± SD. *To assess group differences, we used a one-way ANOVA test. Values in bold show the data group which has mean significantly different from the other two groups.

### 3.2 Multimodal knowledge graph construction and spectral graph convolutions

**Figure 2:**
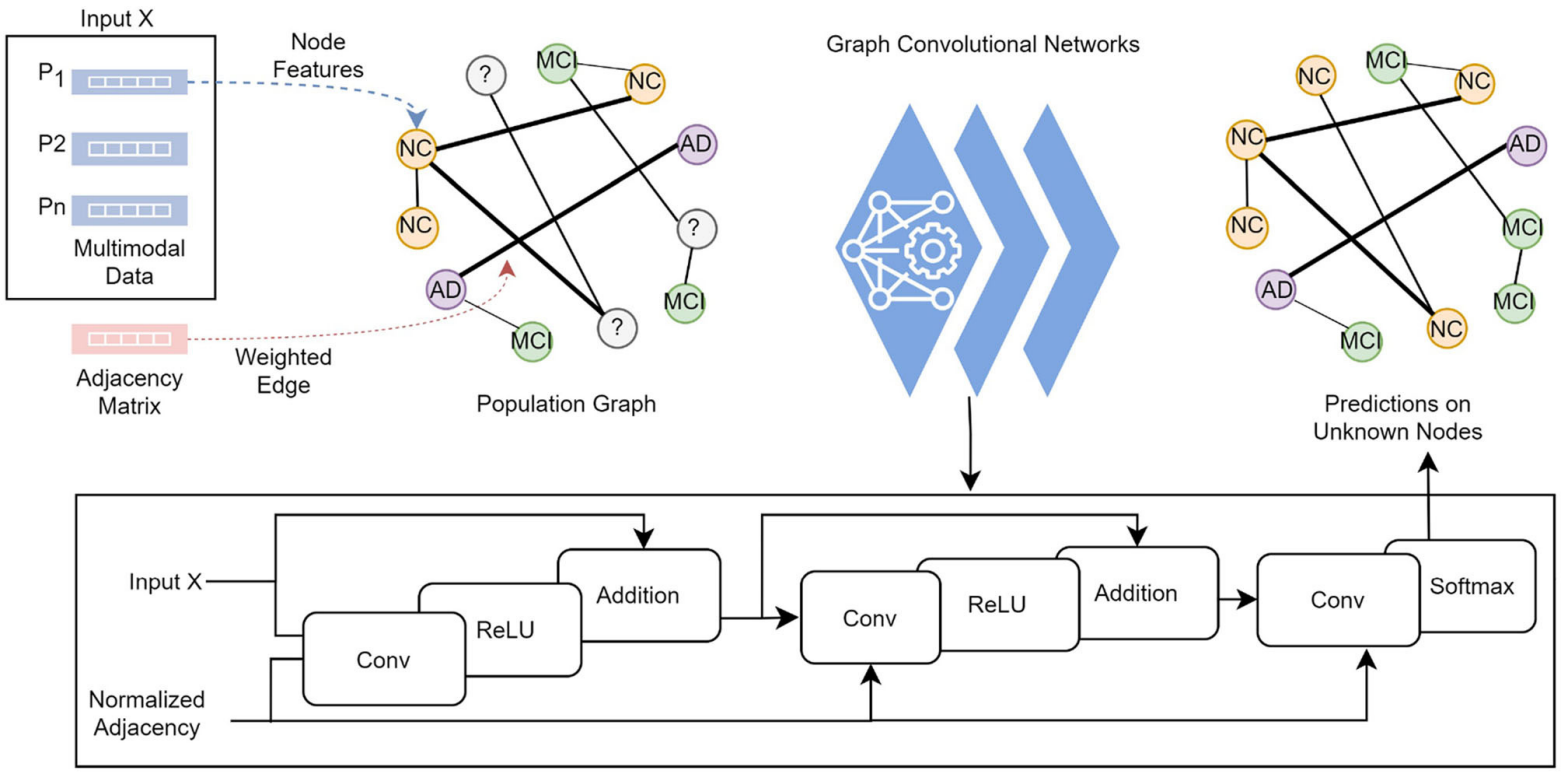
Each subject's feature vector and the pairwise relationships between subjects are modeled as a population graph. The GCN model takes the feature vector and the normalized adjacency matrix and runs through a series of convolutional layers with updated network parameters, ReLU activation function, and adding self-connections followed by a softmax function to output categorical predictions in the final layer.

The knowledge graph is constructed from non-structural data where each subject with corresponding data and the pairwise relationships between subjects are modeled as a population graph. The two main points required to build a population graph are the definition of the feature vector describing each graph node and the connectivity (adjacency) matrix (i.e., edges) weighted based on the similarity between subjects. Figure 2 illustrates the model structure and processes in detail. In our graph, a node contains the demographic, imaging, genetic, cognitive, and neuropsychological test data, which comprises 13 features of a subject. The connectivity matrix provides the information about which nodes are connected and the weight of the connection. The following section describes the computation of the connectivity matrix.

### 3.3 Functional connectivity (adjacency) matrix

The functional connectivity matrix represents the correlation between subjects without any underlying causal model. One way to describe connectivity between subjects is to identify their similarities in their vector space. This can be represented as a graph with an *N*−*by*−*N* adjacency matrix consisting of zeros and ones, where *N* is the number of nodes. If its element is zero, there is no significant similarity between subjects, thereby no edge between two nodes. If its element is 1, there is a high correlation between nodes – node's connection to itself is always zero. Furthermore, the connection between nodes is weighted based on the degree of similarity between subjects by assigning a value between zero and one.

For this study, we have utilized the cognitive test data, including memory, language, and executive function, to construct the functional connectivity matrix. These particular features were selected due to their significant role in the clinical diagnosis of NCs, MCIs, and ADs. Prior research has demonstrated that the connectivity matrix utilizing measures with known links to the pathologies, as opposed to phenotypic information like age and gender, results in better performance (Parisot et al., 2018).

Considering a set of cognitive test measurements (*Mn*), the functional connectivity (adjacency) matrix *A* is defined as follows:


(1) $$\begin{array}{l} A\left(h,w\right)=S\left(h,w\right)\underset{n=1}{\sum }\left(1-\delta \left(Mn\left(h\right),Mn\left(w\right)\right)\right) \end{array}$$

where *S*(*h, w*) is the edge weights computed by a measure of similarity between subjects calculated from δ the Euclidean distance between pairs of observations in cognitive test measures. The distance is a positive scalar value, signifying how far apart the two values are. A smaller distance between two points, *Mn*(*h*) and *Mn*(*w*), indicates a greater similarity. This value is then subtracted from one to represent it as an edge weight. Once this calculation is performed for all measurements, including memory, language, and executive function, we combine and normalize them to determine the final edge weight. Thereby, similar patients are embedded close to each other in an edge-weighted graph, approximating similarity in the network. The objective is to utilize this information to define a proper neighborhood system that optimizes the performance of the graph convolutions.

### 3.4 Spectral graph convolutions

The graph convolutions are computed in the spectral domain (also known as Fourier domain) as the multiplication of an *N*−*by*−*i* feature matrix *X*, where *N* is the number of nodes of the graph, and *i* is the number of features per node with an adjacency matrix *A* that represents the connections between nodes in the graph (Kipf and Welling, 2016). The multiplication operations are weighted with learnable weights. The series of convolutional operations is defined as:


(2) $$H_{l+1}=\sigma_{l}({\hat{D}}^{-1/2}\hat{A}{\hat{D}}^{-1/2}H_{l}W_{l})+H_{l}$$

where:

- σ_*l*_ is the relu activation function.
- *H*_*l*+1_ is *X*.
- *W*_*l*_ is the learnable weight matrix for the multiplication.

The normalized adjacency matrix corresponds to:


(3) $${\hat{D}}^{-1/2}\hat{A}{\hat{D}}^{-1/2}$$

where Â = *A*+*I*_*N*_ is the adjacency matrix *A* of the graph added with the identity matrix *I*_*N*_, and $\hat{D}$ is the degree matrix of Â. The GCN model takes an adjacency matrix *A* and a feature matrix *X* as input and runs through convolutional layers with a ReLU activation function, followed by a softmax function to output categorical predictions in the final layer.

### 3.5 Training and performance of the model

**Figure 3:**
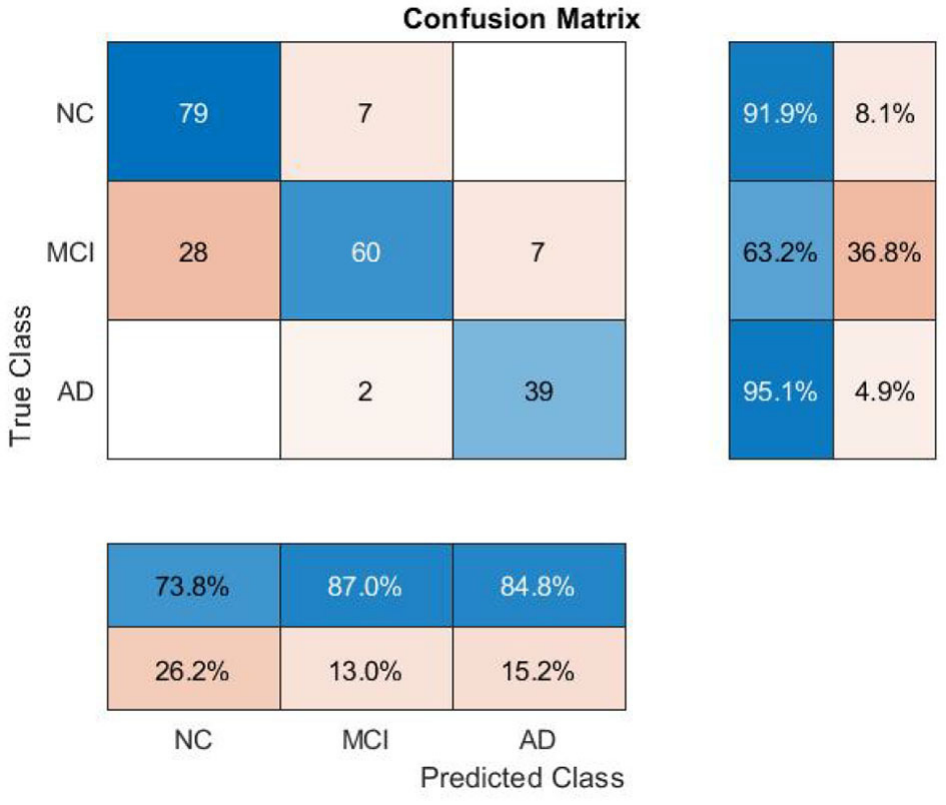
Performance evaluation of the model on unseen data.

The dataset is split into training, validation, and test partitions containing 80%, 10%, and 10% of the data. The sets have been split before the normalization of the data. We train for 100 epochs, set the learning rate for the Adam solver to 0.01, and validate the network after every five epochs. The training loop evaluates the model loss and gradients, updates the network parameters, and validates the network by making predictions on the validation set. The model trained on the ADNI dataset has achieved 78% and 80% classification accuracy on validation and test sets, respectively. The class-wise precision (column) and recall (row) scores show that the AD class has the highest accuracy on average (see Figure 3), as expected, since AD has the most differentiating feature values. The model makes the most incorrect predictions on MCI cases (35 out of 95), which is not surprising as MCI has the most overlapping features among the other two classes.^2^

As highlighted in Table 3, the overall performance of the model when all modalities combined was a multiclass F1 (MCF) score of 81.56% and an accuracy of 80.18%. MCF score is considered a better indicator of the classifier's performance than the standard accuracy measure. Based on the results from different combinations of modalities, we observed variations in the performance between models. In general, the models built without the biomarkers data performed poorly, and these models (D+CT+NT and MRI+CT+NT) improved their performance significantly when combined with the biomarkers data. It has improved overall MCF by 31% and MCA by 27%, particularly for MCI and AD cases. A minor improvement (2% for MCF) was observed when demographic data were included in the model (MRI+CT+NT+BM). In addition, the model trained with all modalities achieved the lowest variance in performance compared to the model built without imaging features. Numerically higher performance was achieved for models where either cognitive or neuropsychological tests were eliminated, but including these modalities did not significantly reduce performance (*p*-value = 0.16). Cognitive and neuropsychological tests are commonly considered in clinical practice though factors such as poor sleep, previous head injury, alcohol etc., might influence these scores. As a caveat, note that these might add noise to the model's overall performance. The model's performance results when features are eliminated individually in each group are shown in Supplementary Table S1.

**Table 3:** Influence of each data group on classification results.

	**Precision %**			**Recall %**			**F1 %**				

**Modalities**	**NC**	**MCI**	**AD**	**NC**	**MCI**	**AD**	**NC**	**MCI**	**AD**	**MCF %**	**MCA %**
All modalities	73.83	86.96	84.78	91.86	63.16	95.12	81.87	73.17	89.66	81.56	80.18
MRI+CT+NT	51.81	62.5	100	100	15.79	25.21	100	78.05	87.67	60.38	59.91
D+CT+NT	48.31	37.5	100	100	6.3	68.29	65.15	10.81	81.16	52.37	54.05
D+MRI+CT+NT	46.74	22.22	100	100	2.11	70.73	63.70	3.84	82.86	50.14	52.70
D+MRI	56.58	76.32	100	100	30.53	78.05	72.27	43.61	87.67	67.85	66.22
CT+NT+BM	55.13	64.1	100	100	26.32	65.85	71.07	37.31	79.41	62.6	62.16
MRI+CT+NT+BM	72.32	91.38	78.85	94.19	55.79	100	81.82	69.28	88.17	79.75	78.83
D+MRI+CT+BM	76.7	89.7	80.39	91.86	64.21	100	83.6	74.84	89.13	82.53	81.53
D+MRI+NT+BM	73.83	88.24	85.11	91.86	63.16	97.56	81.87	73.62	90.91	82.13	80.63
D+CT+NT+BM	71.93	92.98	80.39	95.35	55.79	100	82	69.74	89.13	80.29	79.28

D, Demographics; MRI, Magnetic resonance imaging; CT, Cognitive tests; NT, Neuropsychological tests; BM, Biomarkers and genetic data; MCF, Multiclass F1 score; MCA, Multiclass accuracy.

### 3.6 Explanation method for individual node classification

Interpretability is critical as it can help in informed decision-making during diagnosis and treatment planning. Many methodological advances have been made for medical tasks, such as graph learning, multiple graph scenarios, and graph heterogeneity. GCNs are complex, opaque models which require external means to interpret the model's inner workings. In this section, we describe the explanation method proposed in this work to gain insight into the model's inner workings.

This study adopted a decomposition-based approach for computing the importance score of different input features (Robnik-Šikonja and Kononenko, 2008). We observe the relationship between the features and the predicted value informed by the inner mechanism of the decision-making process. In other words, we examine the output variations concerning the changes in input values. By measuring such variations, we determine the degree of impact on an outcome and reason about the importance of each attribute value. We considered the input features in three levels: individual node features, group-level node features, and edge weights. The proposed method applies the same principle on all three levels.

The model, as a function, maps unlabeled nodes into numerical values *f*:*x*→*f*(*x*). For the node classification task, these numerical values are the probabilities of the class values, *y* is the highest. A node *x* has a known value for each attribute *T*. To measure the effect of each input value, we observe the model's prediction for *x* without the knowledge of event *T*_*i*_ = *t*_*k*_, where *t*_*k*_ is the value of feature *T*_*i*_ for node *x* and the class value *y*. The value of attribute *T*_*i*_ is replaced with an unknown (i.e., NaN) value that does not contain *T*_*i*_'s information. Typically, NaN values are treated as invalid or masked elements and are not considered in the computation. Certain libraries offer a NaN-aware operation function (e.g., “omitnan” in MATLAB) to handle these values in mathematical operations. By utilizing such functionality within the convolutional computations, we obtained the probability for the new input set. We then evaluate the variation between probabilities directly:


(4) $$Var_{i}(y|x)=p(y|x)-p(y|x\setminus \text{​}T_{i})$$

**Figure 4:**
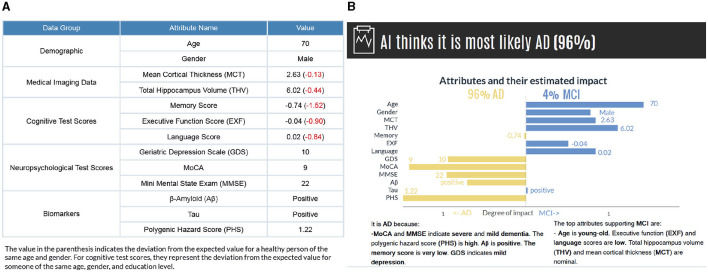
**(A)** The subject's attribute values are presented based on data groups. The values in parenthesis indicate the deviations from a healthy person of the same age and gender. **(B)** The model's prediction and factual and counterfactual explanations. Counterfactual explanations are generated for the second most probable category. While the graph provides a detailed visual representation of how each feature affects the prediction, the text-based explanations describe the reasoning for the influence of each feature.

As a result of this, the decomposition of an input feature brings forth explanations. If the variation between *p*(*y*|*x*) and *p*(*y*|*x*_*i*_) is high, *T*_*i*_ = *t*_*k*_ has a significant influence on the model's prediction; if this variation is low, the influence of *T*_*i*_ = *t*_*k*_ in the prediction is small. Moreover, we consider the direction of the variation, whether *T*_*i*_ = *t*_*k*_ positively or negatively influences the prediction. If the *Var*_*i*_(*y*|*x*)>0, it indicates that the corresponding input feature has a supporting effect on the prediction; if *Var*_*i*_(*y*|*x*) < 0, *T*_*i*_ = *t*_*k*_ has a contrary influence. This direction is observed simultaneously for the second most probable category to examine the effects on the counterfactual case. If the *p*(*y*|*x*) decreases without *T*_*i*_ = *t*_*k*_, it increases the likelihood of another class (or vice-versa), knowing that the probability is distributed among classes. We then present the factual and counterfactual explanations side by side to compare and contrast which attributes contribute to each class (see Figure 4B).

Individual-level explanations are most desirable in the medical field because they could help identify asymptomatic individuals at risk for a future clinical disorder. However, group-level explanations are essential for understanding the overall view of how each modality contributes to the predicted value. Besides generating explanations for each attribute's individual impact, we can also assess their complex complementary dependencies within their data groups. To measure the influence of a combination of input values, we observe the model's prediction for *x* without the input values for *T*_*i* = 1, ..*a*_, where *a* is the number of attributes in a specified group (see Equation 5). Then, the value of attributes for *T*_*i* = 1, ..*a*_ is decomposed concurrently. The variation between probabilities indicates the influence of each data group calculated as:


(5) $$GrpVar_{i=1,..a}(y|x)=p(y|x)-p(y|x\setminus \text{​}T_{i=1,..a})$$

Since we study multimodal data in five categories (demographics, cognitive tests, neuropsychology tests, medical imaging, and biomarker/genetic data), we decompose all attributes in that category and measure the effects on the predicted value. Hereby, we explain the influence of each modality where the change in more than one attribute at once affects the predicted value.

We also considered relational information as graph data contains critical information in edges. To find the influence of neighboring nodes belonging to a certain class *c*, the variation in probability is observed by silencing the edges *E*_*c*_ connecting the node of interest to other nodes. This involved assigning a zero weight to the edges belonging to nodes within a certain class, followed by calculating the variance from the initial prediction value.


(6) $$EdgVar(y|x)=p(y|x)-p(y|x\setminus \text{​}E_{c})$$

In other words, we derived neighborhood-based explanations by methodically removing nodes of a specific class and estimating the influence on the predictions. In medical diagnosis, such explanations are particularly relevant as they provide a norm-referenced evaluation for healthcare practitioners. By comparing a subject to other patients or cases of a similar class, this assessment method helps determine a subject's status within the broader context.

### 3.7 Explanation presentation

We present the results in visual and text form to provide clear and detailed explanations for the end users. Figure 4 illustrates a subject's input values along with the model's prediction and explanations for individual features. The horizontal axis shows the attributes' estimated impact. Considering that the evaluation of probabilities could have a higher cognitive load, we have normalized the influence values to increase comprehensibility. The vertical axis contains the names of attributes and their values for the selected instance attached to the bar. The length of the bars corresponds to the impact of the attribute values in the model expressed as Equation 4. By comparing the bar length, the user gains insight into the degree of impact of each feature value. The attributes contributing to the actual class are on the left-hand side, and the counterfactual case (i.e., the class with the second-highest probability) is on the right-hand side. It is, in fact, possible to generate counterfactual explanations for all other classes regardless of the probability values.

The visual explanations are accompanied by text-based reasoning, which describes why certain attributes contribute to an outcome. These explanations are complementary to increase the comprehensibility of the visual explanations. We discretized the features in different categories to generate text-based explanations. The general cut-offs are known for some features [e.g., GDS (0 < normal < 9, 10 < mild depressive < 19, 20 < severe depressive), MMSE (0 < severe dementia < 9, 10 < moderate dementia < 20, 21 < mild dementia < 24, 25 < normal cognition < 30)]. For the remaining features, we grouped continuous data into the set of bins (very low, low, nominal, not so high, high, and very high) based on the distance from the mean, measured in standard deviations considering all the categories (NC, MCI, and AD) together (see Supplementary Figure S2 and Supplementary Table S2). Also, to make the diagnostic procedure easier for a user in our human-subject evaluations, we have calculated how these values deviate from a healthy person of the same age and gender and present them in parenthesis (Figure 4A). For cognitive test scores, they represent the deviation from the expected value for someone of the same age, gender, and education level.

**Figure 5:**
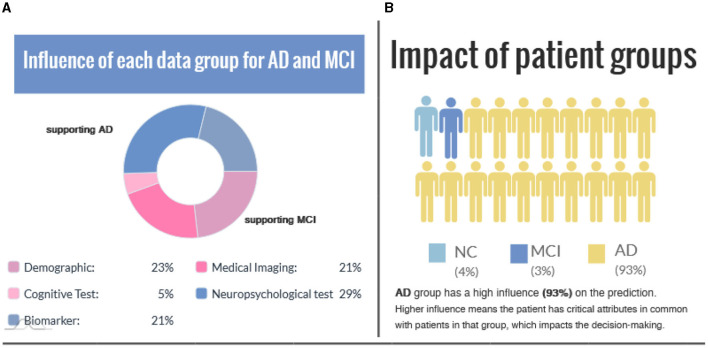
**(A)** The influence of each data group is represented for the factual and counterfactual cases. **(B)** Impact of patient groups indicates the similarity between the case (i.e., the interest point) and patient groups.

Figure 5 shows group-level explanations and the impact of patient groups based on feature similarity. Figure 5A visualizes supporting and opposing data groups depending on the influence on the predicted value. The pie chart provides a brief overview of the relative proportions of supporting features within each class, and color coding is incorporated to distinguish between factual and counterfactual cases. An alternative visualization of this can be found in Supplementary Figure S3. Figure 5B illustrates the impact of patient groups based on the percentage of subjects with similar features connected in the graph. A higher percentage value means the patient has critical attributes in common with those in that group, impacting the decision-making.

## 4 Results

In order to evaluate the proposed explanation method, we first measured a functional property of the explanation. Our evaluation aims to demonstrate the stability of the explanations as to whether it shows consistent results concerning neighborhood information for subjects with similar attribute values. We also conducted a comparative study with Shapely Addictive exPlanations (SHAP) to assess how comparable the explanations generated by both methods are. We then conducted a human subject study to assess the correctness, predictability, and quality of the explanation and whether it facilitates understandability and trust in the model.

### 4.1 Stability of the explanations

Stability compares explanations between similar cases for a specified model. Higher stability indicates that subtle variations in the feature values of an instance do not significantly change the explanation unless these minor changes also alter the prediction. To measure the stability, instead of slightly varying a feature value, we identified the similar nodes using the edge weights, considering that higher edge weights signify higher patient feature similarity. It would give us slight variations in feature values for patients in the same class. Also, identifying the samples this way would illustrate the real-world similarities and variations in actual patient data. As a result, we measure the stability of the explanations based on neighborhood explanation similarity.

**Figure 6:**
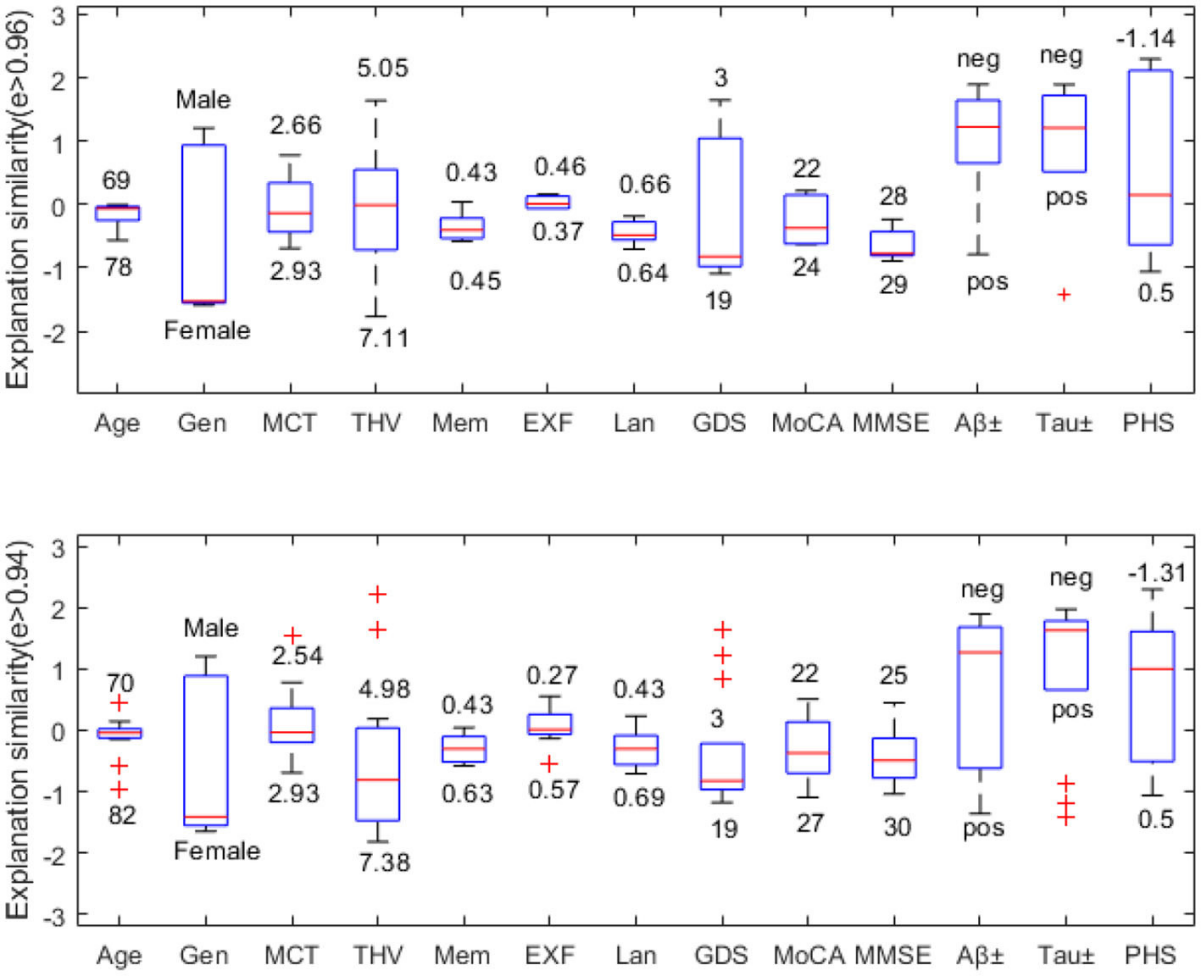
Neighborhood explanation similarity. Box labels show the highest and lowest attribute values within that neighborhood identified based on the edge weight.

In this example, we choose a challenging MCI case since MCI has the most overlapping feature values to both NC and AD classes. Figure 6 compares explanation similarity for samples in a different proximity. The first plot examines the nodes with edge weights e > 0.96. This neighborhood contains five nodes, while the second plot observes the nodes with edge weights e > 0.94, which includes 13 nodes belonging to the MCI class. Given that edge weights fall between the spectrum of zero and one, these particular points may be classified as residing in the same vicinity but may possess greater differences in specific features. These points have been chosen to demonstrate how node features vary within “the same neighborhood” and how each attribute's impact fluctuates in this particular context.

For instance, when it comes to categorical values such as gender or biomarker positivity (e.g., male-female, positive-negative), there tend to be more discrepancies. Consequently, there are higher fluctuations in the resulting feature importance values. However, when examining the cognitive test features of nodes with e > 0.96, we see that memory falls between 0.43-0.45, executive function between 0.37–0.46, and language between 0.64–0.66. Similarly, nodes with e > 0.94 show memory scores between 0.43-0.63, executive function between 0.27–0.57, and language between 0.43–0.69. Therefore, we can see that feature importance values tend to fall within the same range (see Figure 6).

We can also assess how much feature importance varies by looking at where it falls in the influence area. Feature importance values located above zero are considered supporting, while those below zero are opposing. Narrow boxes generally indicate stable feature importance values. While data points with (e > 0.94) demonstrate more outliers than those with (e > 0.96), generally, they remain within the same influence area. Figure 6 reveals that MMSE has consistent feature importance values, while age, THV, GDS, and PHS exhibit proportional variation based on changes in the input space.

In order to quantify the similarity of the feature importance values, we define the distance between the highest and the lowest influence values for the nodes in different proximity. For instance, for the MCI case (node), five neighboring nodes are connected by edge weights e > 0.96. We evaluate the feature importance of each node and determine the absolute value of the Euclidean distance between the minimum and maximum feature importance values for each attribute across these nodes. Table 4 shows how similar the explanations are on different levels of edge weights, where the smaller distance values suggest similar feature importance values. It is worth noting that categorical values, Gen, Aβ±, and Tau±, exhibit the greatest variation in feature importance values. Additionally, as the edge weights decrease, the number of neighboring nodes belonging to the same class increases twofold, thereby expanding the feature space. This could be the reason for the lower distance values observed for certain features, such as MCT and THV, at lower edge weights.

**Table 4:** Explanation similarity measured by the Euclidean distance between minimum and maximal influence values.

	**Age**	**Gen**	**MCT**	**THV**	**Mem**	**Exf**	**Lan**	**GDS**	**MoCA**	**MMSE**	**Aβ±**	**Tau±**	**PHS**
*e* > 0.96	0.01	0.89	0.22	0.42	0.04	0	0.03	0.71	0.22	0.07	0.31	0.39	1.0
*e* > 0.94	0	1.0	0.17	0.59	0.11	0.07	0.14	0.26	0.29	0.21	0.94	.42	0.85
*e* > 0.90	0.04	0.93	0.01	0.24	0	0.11	0.12	0.04	0.14	0.07	0.87	1.0	0.85
*e* > 0.85	0.08	0.91	0	0.19	0.05	0.06	0.17	0.47	0.18	0.06	0.76	1.0	0.57
*e* > 0.80	0.19	0.89	0	0.29	0.07	0.05	0.22	0.66	0.15	0.04	0.77	1.0	0.37

The smaller distance values suggest similar feature importance values for that particular feature.

### 4.2 Comparative evaluation with Shapely Addictive exPlanations (SHAP)

In this section, we compare our explanation results with the SHAP (Shapely Addictive exPlanations) by estimating the contribution of individual features to a prediction at the specified interest point (i.e., a particular patient data to be predicted). SHAP is a local model-agnostic post-hoc explanation method based on the Shapley value concept from game theory (Lundberg and Lee, 2017; Lundberg et al., 2020). It is a widely adopted explanation method in AD disease diagnosis-related tasks to evaluate the feature's influence on the prediction (see Table 1).

The Shapley value of a feature for an interest point explains the deviation of prediction for the interest point from the mean prediction when the values in the training set replace the current feature values. Thus, for each interest point, the Shapley values approximate the impact of the variation of the prediction from the mean prediction. One of the limitations is that the computation can be slow when the predictor has a high number of observations. Although one might also choose to use a smaller sample of the training set for faster computation, we used the entire training set in our experiments.

**Figure 7:**
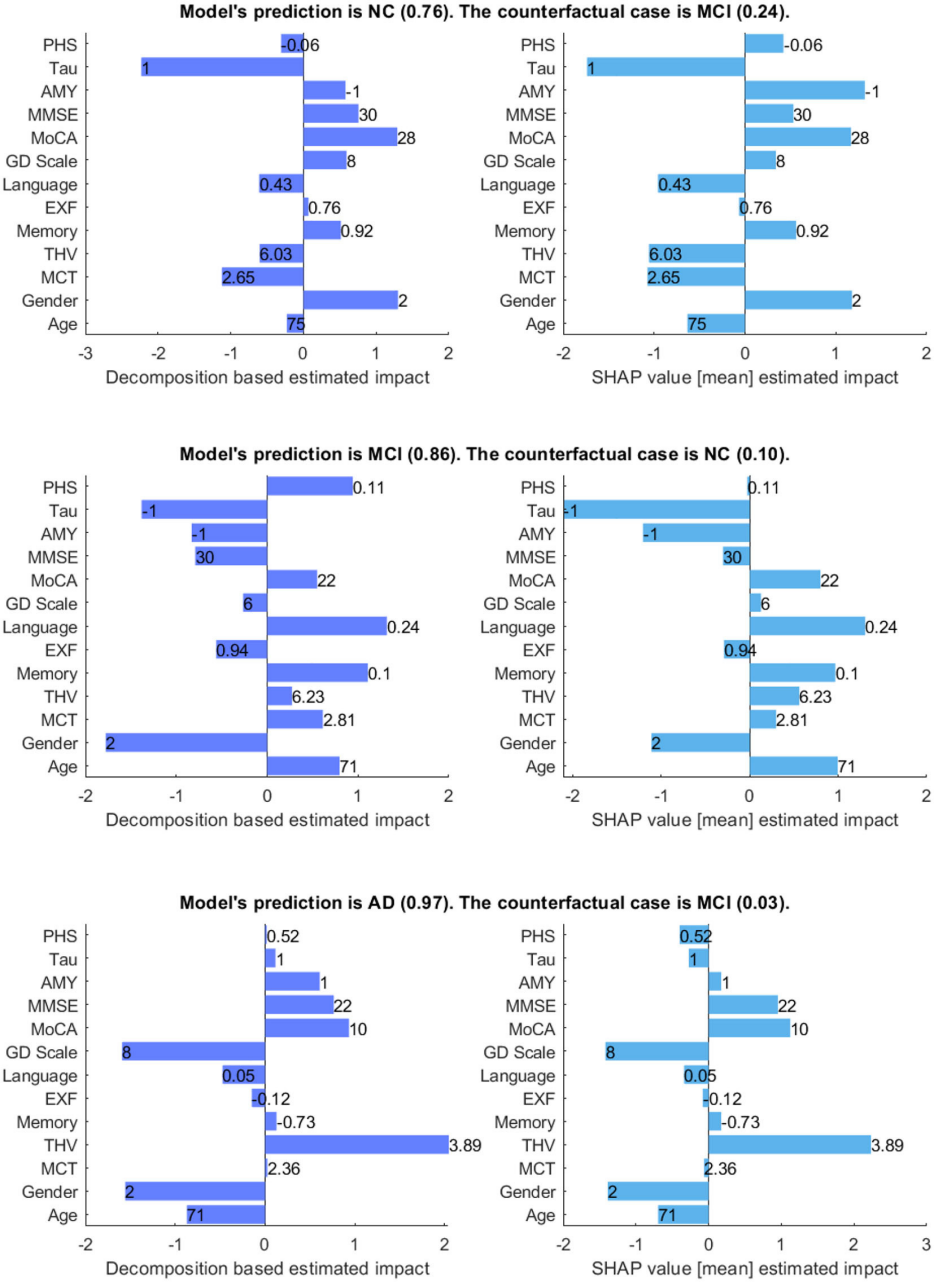
Comparison between decomposition-based explanations and SHAP value estimated impact. The bar length indicates the average degree of impact of the feature value, and bar labels show the feature value. Aβ&TAU: Positive (1), Negative (-1), Gender: Male (1), Female (2).

Figure 7 shows the model's prediction, and the estimated impact of each feature on the prediction for three randomly selected interest points, comparing our method and SHAP values. In the plots, the direction of the bar indicates whether a feature has a supporting or opposing effect on the prediction. The positive values represent features that increase the prediction probability of the class, while the negative values are features that decrease the probability of the class. The bar length indicates the average degree of impact of the feature value, and bar labels show the feature value.

Generally, both explanation methods agree on the direction of impact of each feature with slight variations in the value of feature importance for given scenarios. For the NC case, both explanation methods show Gender (Ours = 1.3 vs. SHAP = 1.18) and MoCA (Ours = 1.3 vs. SHAP = 1.17) as the most contributing features and TAU (Ours = -2.23 vs. SHAP = -1.74) as the highest opposing feature, which is not surprising given that its status is positive. Besides, both show Aβ, MMSE, GDS, and memory as positive influences for the prediction; Aβ is negative, and all other features are within the normal range for the healthy group. The imaging data negatively influences the prediction, as shown by both methods. There is a main disagreement on the Polygenic Hazard Score (PHS) in which SHAP indicates it as a contributing feature though it is “not so high” for the NC class (can be seen in Supplementary Table S2). This difference seems to persist in other scenarios as well.

For the MCI case, both methods show that the model mainly relies on age (Ours = 0.94 vs. SHAP = 0.99), memory (Ours = 1.11 vs. SHAP = 0.97), language (Ours = 1.32 vs. SHAP = 1.30) and MoCA (Ours = 0.55 vs. SHAP = 0.79) to make the prediction. The imaging data is also shown as contributing features, as the feature values are “not so high” for a healthy person of the same age and gender. In contrast to SHAP, our method shows PHS as a contributing feature, given that this feature is “high”. Both methods show Aβ and TAU as opposing features, as expected since both are negative. Besides, MMSE and EXF are given as negatively influencing the prediction class MCI, which is predictable considering both are within the normal cognition range.

Concerning the AD case, both explanation methods show that THV (Ours = 2.05 vs. SHAP = 2.23), MoCA (Ours = 0.93 vs. SHAP = 1.12), and MMSE (Ours = 0.76 vs. SHAP = 0.95) values play a significant role in making the prediction. Aβ (Ours = 0.6 vs. SHAP = 0.17) is also shown as a positively influencing feature since its value is positive. For this given scenario, it appears that EXF, memory, and PHS have less impact on the probability of the class, regardless of the direction of the influence. Surprisingly, SHAP indicates TAU(+) as an opposing feature for the predicted class AD with a low estimated impact.

**Figure 8:**
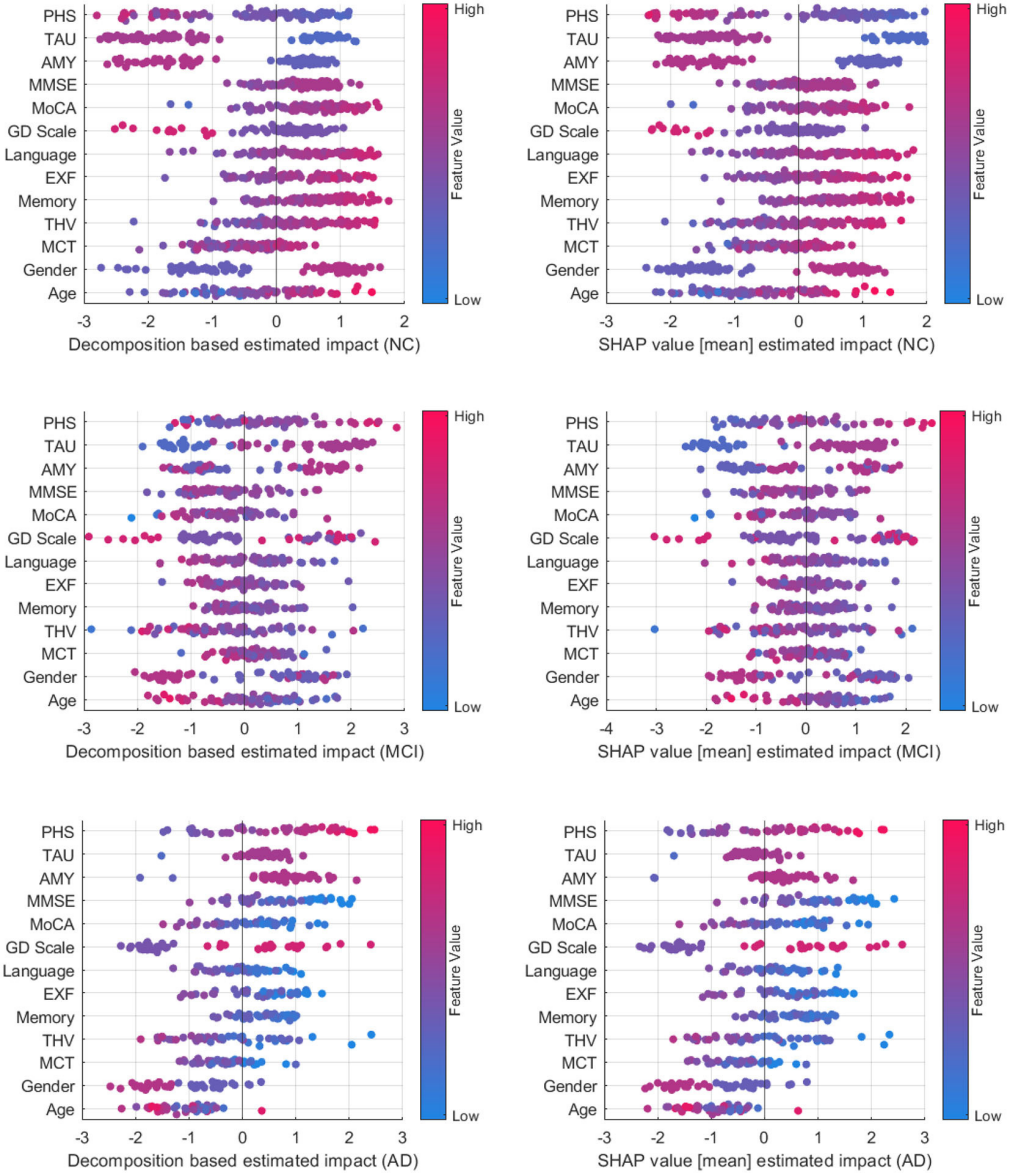
Comparison between decomposition-based explanations and SHAP value estimated impact over all test set.

Figure 8 compares decomposition-based explanations and SHAP value estimated impact across the test set. Overall, the explanation results obtained from the decomposition-based method are comparable to the SHAP explanations, given that the correlation coefficients between pairs of feature importance reveal high correlations (NC = 0.919, MCI = 0.938, and AD = 0.939). The figure shows that both explanation methods agreed that as the feature values for imaging data, cognitive test results, and the MMSE and MoCA scores grow, the positive influence of these features increases for the NC class. The opposite effect is observed for MCI and AD cases where lower feature values contribute to these classes. Similarly, the lower polygenic hazard score with negative Aβ and TAU has a favorable influence on the NC class and a reverse effect on AD and MCI classes. Both methods show similar patterns for age and gender as well across all samples. While the female gender appears as a supporting feature for NC cases, it has the opposite effect on MCI. For AD cases, the female has a higher negative impact on the predictions than the male, as demonstrated by both methods.

A class-wise comparison shows both explanation methods captured the distinct effect of features on NC and AD cases more explicitly than on MCI cases. In feature-wise evaluation, slight differences between estimated feature importance might be due to the variations between observations affecting the overall estimated impact (mean) for SHAP values. Compared to SHAP, one advantage of our approach is that it is computationally more efficient. On average, the computation time to generate explanations for an instance with the decomposition method is 2.41s, whereas SHAP performed a mean of 52.3s to produce explanations for the same examples. Although both methods are model agnostic, making them widely applicable across various machine learning models, SHAP explanations tend to be more complex mathematically due to their consideration of feature interactions. On the other hand, the decomposition method is more straightforward and intuitive. Yet, the decomposition-based method can also be computed for more than one feature, as shown in group-level explanations. This offers a way of understanding how multiple features interact to influence predictions, particularly in complex models. Both approaches have their strengths and are suitable for different contexts where one method may be more effective than the other in providing insightful explanations. Ultimately, the choice of method depends on the interpretability needs and the complexity of feature interactions.

### 4.3 Human-grounded evaluation with domain experts

We conducted a survey to evaluate the domain expert's agreement on the model's prediction and rationale, the predictability of the model, and the quality of explanations for justifying a model's prediction. Eleven experts who work and research in the Alzheimer's disease-related domain in Sweden participated in the survey. The survey includes four sections: demographics, diagnostic study, predictability, and general questions.^3^ The details of each section and a summary of the results are as follows:

#### 4.3.1 Demographics

This section explores the participants' backgrounds, including age group, occupation, and years of expertise. 42% of the participants are over 54 years old with more than 11 years of experience in the domain. They indicated their profession as geriatricians, psychogeriatricians, general practitioners, neuroradiologists, neuropsychologists, and researchers working on brain aging, Alzheimer's disease, and dementia.

#### 4.3.2 Diagnostic Study

We selected five correctly predicted scenarios (two ADs, two MCIs, and an NC) and generated explanations for each case. Participants were presented with attribute values in the format shown in Figure 4A and asked to predict the person's diagnosis and indicate their confidence level on a 10-point Likert scale from one (not confident) to ten (very confident). The next task for the participant was to judge the AI's prediction and rationale, as presented in Figure 4B. They were asked to rate their agreement with the AI's prediction and rationale and explain their rating–why they agreed or disagreed with it.

Concerning the first question, the ratio of correct predictions to wrong predictions made by participants was 43 to 12. While all the participants correctly predicted the AD patients, most were mistaken for the MCI subjects with AD and NC. Supplementary Table S3 shows the confusion matrix on the domain expert's prediction.

**Figure 9:**
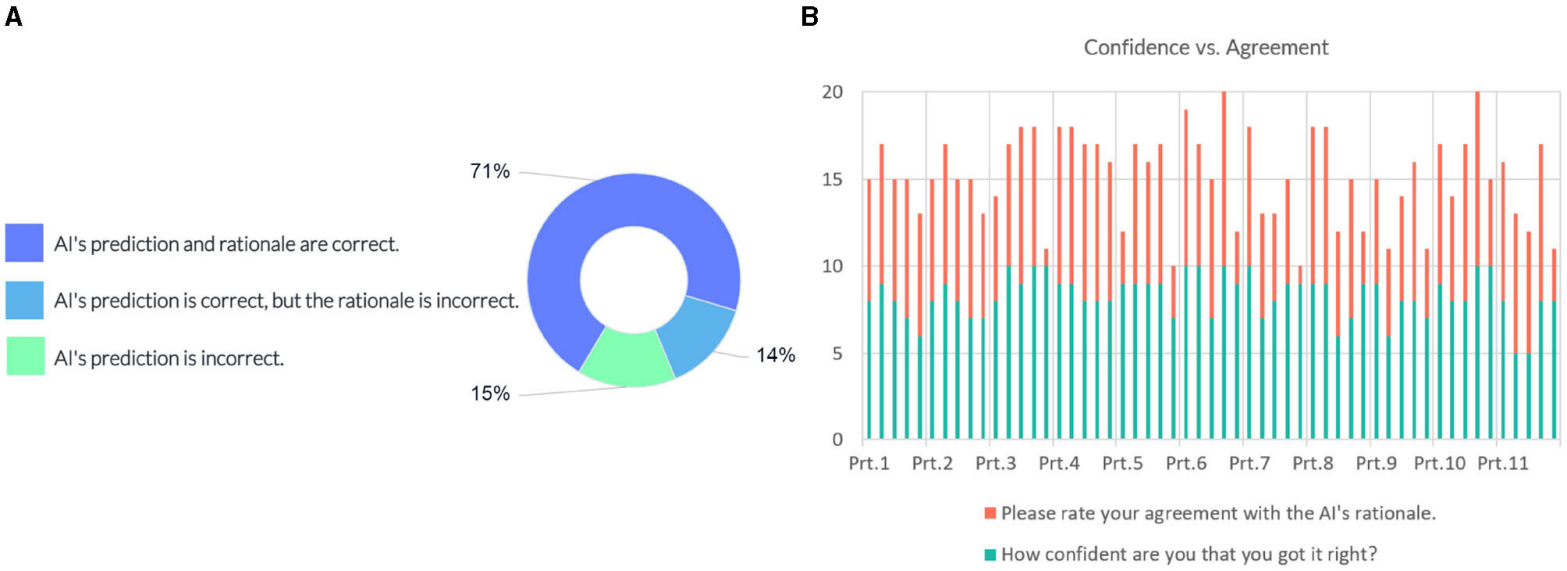
**(A)** Domain expert's judgement on the AI model's prediction and rationale. **(B)** Instance-wise comparison between the participants' (Prt.) confidence and their degree of agreement on the model's rationale.

The following question investigates whether the model's prediction and explanation are acceptable to domain experts (see Figure 9A). 71% of the responses indicated that the AI's prediction and rationale are correct, 14% of answers suggested that the prediction is correct, but the underlying reasons are incorrect, and 15% of the responses considered the model's prediction incorrect.

We compared the confidence of the individual participants against their degree of agreement with the justification provided by the model. Figure 9B shows scores in intervals devoted to each participant, showing their responses for each instance. Most participants gave a confidence score above six on the 10-point scale, indicating a high degree of certainty in their judgment of the case. Eight out of the participants' 12 incorrect predictions rated “AI's prediction is incorrect”. The comparison with the agreement scores shows that overall they conform with the model's rationale when the model's prediction and rationale match theirs; participants disagree with the model's prediction when their prediction does not match the model's prediction.

In some instances, participants gave a higher score on agreement than their confidence level. A couple of responses in regard to a higher agreement score given by the participants:

“*I agree with MCI because we cannot ignore MoCA of 22.”*—P6

“*My idea almost got along with the AI rationale.”*—P8

“*Agree because of the statistics is in favor of MCI from my clinical point of view.”*—P10

“*I agree and find the result to be supporting when making a clinical judgment.”*—P11

Where the participants disagreed with the AI's prediction and rationale, they commented that:

“*Age is not so much important to differ MCI from AD.”*—P5

“*I think it's AD since all biomarkers are positive and MoCA is very low. MMSE is probably just about to follow, but I believe it's the early stage of a rapid AD.”*—P6

“*It is MCI because the gender is male? I do not understand the AI's rationale.”*—P9

#### 4.3.3 Predictability

After the participants had engaged with the AI's prediction and rationale and gotten an understanding of how it reached a decision, we investigated whether participants could anticipate what the model would predict for a particular set of attribute values. We presented participants with five new cases (two ADs, two MCIs, and an NC) and asked their opinion on how the AI might diagnose the case. Then, they were asked to indicate in a free text which top features AI might rely on and their agreement with this rationale on a scale from one (strongly disagree) to ten (strongly agree).

The ratio of correct to wrong presumption was 35 to 20. Again, AD has the highest correct prediction rate, while MCI and NC cases are often mistaken for each other (see Supplementary Table S4 for confusion matrix on the domain expert's presumption of the prediction).

Next, we analyzed how well the domain expert's mental model of the system matches the actual rationale of the AI model. We carried out this analysis on where the participants were right about their assumption of the model's prediction (35 responses). Table 5 summarizes the comparison between feature groups the AI model relies on and the domain expert's estimation of the AI's rationale. Since the responses were in free text form, we counted the number of participants who indicated a certain data group (or a feature from that group) supporting or opposing the model's prediction. Overall, there is a high consensus for AD cases where neuropsychological tests and biomarkers are presumed to be the supporting feature groups in which the participants were right. One of the participants' responses shows that they recognize the model weighs higher on the demographic data at times. The participants who provided demographics as contributing feature group expressed that:

“*AI might rely on age and GDS.”*—P8

“*AI may mainly use gender and tau.”*—P8

**Table 5:** Comparison between feature groups the AI model relies on and the expert's estimation of the AI's rationale.

		**Case 1 (MCI)**		**Case 2 (MCI)**		**Case 3 (AD)**		**Case 4 (AD)**		**Case 5 (NC)**	

		**Actual**	**Presumed**	**Actual**	**Presumed**	**Actual**	**Presumed**	**Actual**	**Presumed**	**Actual**	**Presumed**
Demographic		(+) 21%	(+) 1	(+) < 1%	(+) 1	(-) 29%	0	(-) 34%	(-) 1	(+) 12%	(+) 1
Medical Imaging:		(-) 4%	(-) 1	(+) 23%	0	(-) 21%	(+) 2	(-) 8%	(+) 2	(-) 25%	(+) 1
Cognitive Test:		(-) 11%	(+) 2	(+) 27%	(+) 2	(+) < 1%	(+) 4	(-) 8%	(+) 3	(+) 1%	(+) 1
NT:		(-) 35%	(+) 2	(-) 29%	(+) 1	(+) 31%	(+) 5	(+) 20%	(+) 7	(+) 37%	(+) 3
Biomarker:		(+) 29%	(+) 4	(-) 21%	(-) 1	(+) 18%	(+) 7	(+) 30%	(+) 8	(-) 25%	(-) 2

Supporting (+) and opposing (-) feature groups for the prediction are given along with the estimated impact (in percentage), signifying the degree of influence of the feature group for the actual model. For the presumed section, values show the number of participants who indicated that the feature(s) the AI model might take into account positively (+) or negatively (-).

It is also interesting to see that two participants indicated neuropsychological tests as contributing to MCI (Case 1), while the model does not count for any of the features in this group. Similarly, in Cases 3, 4, and 5, participants' responses suggest that participants might think that AI relies too often on medical imaging data in general.

**Figure 10:**
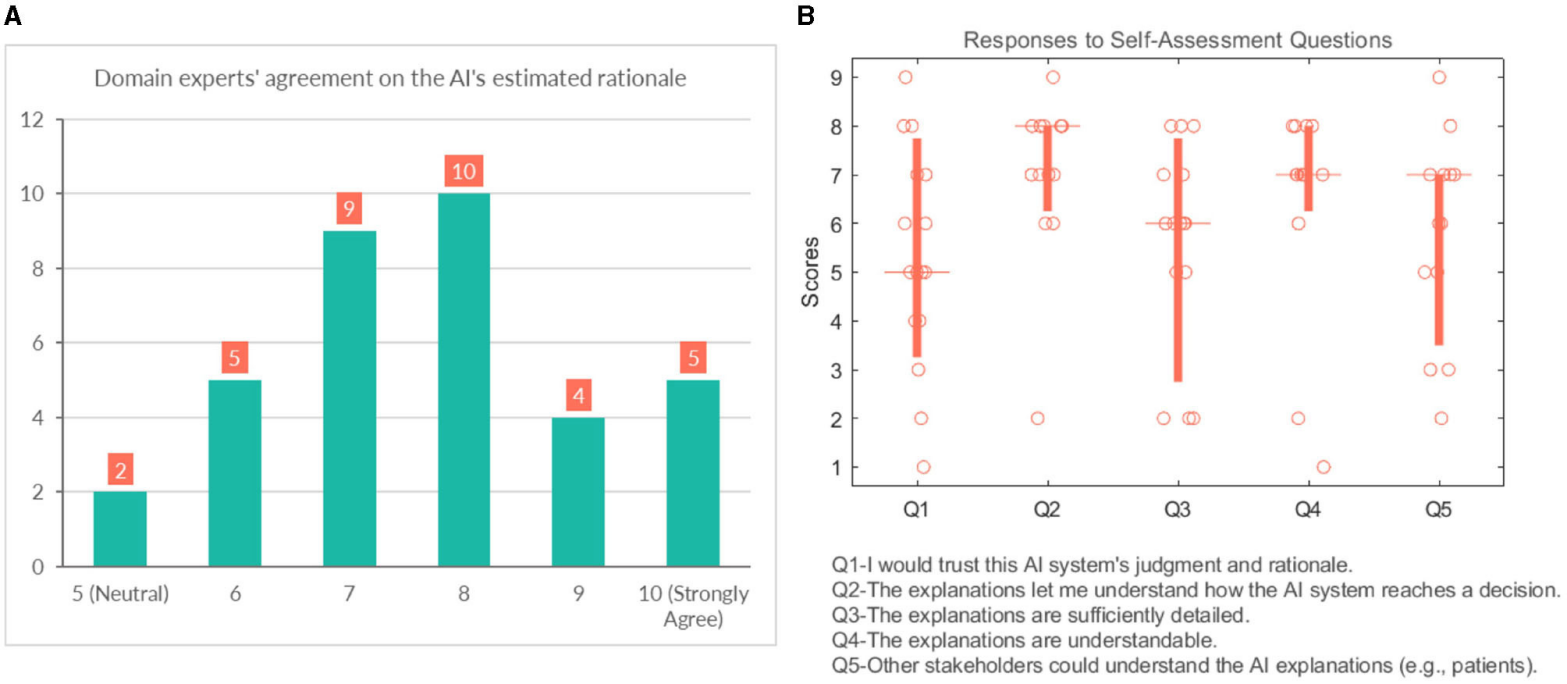
**(A)** Domain experts' level of agreement on the rationale ascribed to the AI model. **(B)** Domain experts' responses to self-assessment questions.

We also asked them to rate their agreement on the hypothetical rationale they projected onto the AI model to assess whether the participant's mental model of the system matches their judgment of the case. Participants gave a score between 5 (Neutral) and 10 (strongly agree) on the 10-point Likert scale, which shows general agreement on their assumptions, as shown in Figure 10A.

#### 4.3.4 General questions

This section analyses several self-reported subjective measures regarding trust, quality of explanations, and explanation satisfaction (see Figure 10B). Regarding the first question (Q1-“I would trust this AI system's judgment and rationale.”), the responses were rather diverse and polarized at times (median = 5). With respect to understandability, most participants would agree that the explanations allow them to understand how the AI system reaches a decision (median = 8). It is also in line with the results that participants believed that “Q4-The explanations are understandable.” (median = 7). The picture remains undecided and even shows an element of polarization concerning Q3 (“The explanations are sufficiently detailed.”) (median = 6). They were also divided with their responses when asked whether other stakeholders could understand the AI explanations (e.g., patient, patient's family, other caretakers) (median = 7).

Overall the self-assessment results suggest that explanations are clear and help the participants to understand the model; however, the explanations could use more popular science terminology to make them more accessible to other stakeholders.

“*It would be good to use different terminology for the different features (more appropriate and clearer for lay persons e.g., abnormal instead of positive amyloid, explain/rephrase terms like polygenic hazard score).”*—P7

## 5 Discussion

In this work, we presented a GCN model for multimodal data in a node classification task for AD diagnosis and provided a decomposition-based method to explain predictions made by the model. Although GCNs have been proposed for AD-related diagnosis tasks from disease diagnosis to detection (Zhou et al., 2022; Lei et al., 2023), this is the first study considering explanations for the model's prediction for clinicians. Studies have presented explanation methods for other machine learning models for AD-related tasks (Kamal et al., 2021; Bogdanovic et al., 2022; Mulyadi et al., 2022), as shown in related work. In contrast to these works, we presented feature importance not only at the individual level but also at the group level and the neighborhood level, which provided additional information to support the classification of each case. Furthermore, we presented individual and group-level explanations in factual and counterfactual pairs. Counterfactual explanations give insight into what would have been the explanation if the case was other than the predicted class, indicating which features contribute to the contrasting class. The visual explanations are followed by descriptive text indicating why specific attributes contribute to an outcome. These explanations complement graphic plots by verbalizing which range a feature value falls.

In contrast to prior research, we introduced a comprehensive assessment approach incorporating both objective and human-centered evaluations. Our analysis revealed the explanations' stability, with consistent outcomes concerning minor changes in input values. In a comparative investigation with the SHAP explanation technique, we observed similar patterns in resulting explanations; nevertheless, our decomposition-based methodology exhibited significantly faster computational speed.

We conducted a thorough evaluation of user-XAI interaction, examining key factors such as explanation accuracy, model predictability, trustworthiness, and explanation quality. Through analyzing their responses to the model's predictions and the underlying reasoning, we found that the explanations provided by the model for accurate predictions were confirmed by domain experts. In fact, 71% of responses verified the model's predictions and rationale as correct. We acknowledge that this percentage might have been different if participants had also seen explanations for incorrectly classified cases. This assessment mainly focuses on measuring the accuracy of explanations for correctly classified cases through expert opinion to verify the consistency of the explanations with the domain knowledge. There is a risk of misleading the user since explanations for the misclassified cases might still look rational. Although it would be beneficial to investigate the potential deceiving qualities of explanations for both correct and misclassified cases, this is beyond the scope of our current work.

Regarding the features used, it's possible that they may not provide enough information for clinicians to differentiate between MCI with AD and NC cases. In the clinical diagnostic procedures, they may rely on other findings such as information about a person's ADL (Activities of Daily Living), data from an informant (e.g., spouse, relative, friend), education, comorbidity, and medication, such classification can be challenging even in in-person assessment in clinical practice. It might be the reason for the misclassifications, specifically in MCI cases made by the experts. It resulted in a certain level of disagreement on the model's prediction and rationale. Some participants disagreed with the model's rationale when age, gender, and imaging data are shown as high influencing factors, as commented:

“*AD is a clinical diagnosis and cannot be established by neuroradiology alone. The AI estimates rely too much on THV.”*—P9

On another note, the proposed framework could benefit from different graph construction strategies where these features are weighted in the model appropriately to overcome this limitation. One possible way could be incorporating them as edge features proposed by Parisot et al. (2018). The weights could be derived by asking experts how much they would regard each data type in their diagnostic procedures. That way, e.g., demographic information might be down-weighted. The results could potentially increase the trustworthiness for the clinicians. We also acknowledge that the classification results from the model are likely driven by the specifics of the dataset, and more generalizable results might be obtained if the model was trained on several, more diverse data sets. For instance, in our dataset, MCI were younger on average, which may contribute to the classification, whereas that is not a given that would be the case in every sample.

Concerning the model's predictability, the participant's beliefs about the model's over-reliance on demographics and neuroimaging data are reflected in their responses. While it was correct for the demographic data concerning the imaging data, it showed a faulty model belief where the model did not account as strongly for imaging features as anticipated for the given examples. In this part of the study, we have only assessed their agreement with their assumption of the model. One could also consider further investigating the participants' reaction to actual explanations generated by the model and if it leads to updating the user's mental model of the system. Considering that some participants ranked rather low in trusting the AI model's judgment and rationale, updating the user's mental model might help build more appropriate trust in the model. It might also increase decision supportiveness, and human accuracy as the user would be more certain about the model's strengths and limitations.

The results from the self-assessment questions show that the explanations provided made the audience understand the reasoning behind a prediction. There was no consensus on the presentation of the explanations being sufficiently detailed, pointing to the subjectivity of the matter. It highlights the need to adapt the explanation to the audience and the capability to provide detailed explanations in response to a request. For more comprehensive explanations, further studies might consider clinical workflow integration (i.e., history, symptom duration, and development) and investigate how a clinical expert could refine a model decision. A human-in-the-loop process is necessary to increase the usability of such systems by clinicians to support decision-making. Future work could consider extending the model to a more clinically realistic situation by, e.g., incorporating additional dementia types (such as vascular dementia, frontotemporal dementia, Lewy Body Dementia, etc.) or other reasons for cognitive impairment (e.g., strokes, medication side effects, substance abuse, vitamin deficiency, etc.). That would, indeed, require additional pieces of information (features) to be incorporated into the model. Nonetheless, high-quality open-access datasets with that level of detail are not easily available, and the current work used ADNI as a first step to develop the methodology used. However, as the next validation step, the transferability of the current results could be tested in other similar AD databases [e.g., The Australian Imaging, Biomarker and Lifestyle Flagship Study of Aging (AIBL)].

Finally, it is worth noting the unique advantages and challenges that come with GCN models. While they excel in modeling complex, multimodal data, their optimal performance often necessitates substantial amounts of graph-structured data. In contrast, traditional models like XGBoost might perform better in scenarios with smaller datasets or lacking explicit graph structures. Moreover, GCN models typically require fine-tuning hyperparameters related to graph structures and neighborhood sampling, rendering them more sensitive to hyperparameter choices. Nevertheless, the efficacy of GCNs lies in their ability to incorporate information from neighborhood nodes and leverage local connectivity patterns within the graph. Mitigating hyperparameter sensitivity in GCNs can be approached through strategies such as sensitivity analysis, transfer learning, and leveraging domain-specific knowledge.

## 6 Conclusion

We presented a decomposition-based explanation method for the GCN model trained on the ADNI dataset, including various aspects of diagnostic procedures. Explanations show how each feature from different modalities contributes to a diagnostic result in multiple levels of detail. Functional evaluation of the proposed explanation method analyzing the stability of the explanations suggests that the similarity of explanation generated in neighboring nodes depends on the degree of edge weights. The nodes with higher edge weights produced higher similarity in explanations. Evaluating the explanations in a user study with domain experts, we observed that participants agreed that the explanations presented were clear and helped to understand the model's predictions. The feedback we have received suggests that an explainable AI model for diagnosing AD could be a valuable instrument to summarize an individual's clinical findings and participate in the process of making a diagnosis. However, the model needs potential improvements to incorporate before adoption into clinical practice, such as weighting different types of data, informed by domain experts and incorporating additional features, such as activities of daily living and informant questionnaires, which might also promote clinicians' trust in the model. Nevertheless, the features included in our model are similar to previous approaches in the literature, and our explanation method is comparable to SHAP values with significantly reduced computational time.

## Data availability statement

Publicly available datasets were analyzed in this study. This data can be found here: Alzheimer's Disease Neuroimaging Initiative (ADNI) database (adni.loni.usc.edu).

## Author contributions

ST: Conceptualization, Data curation, Formal analysis, Funding acquisition, Investigation, Methodology, Resources, Software, Visualization, Writing – original draft, Writing – review & editing. SP: Supervision, Writing – review & editing.

## Footnotes

^1^Data used in the preparation of this article were obtained from the Alzheimer's Disease Neuroimaging Initiative (ADNI) database (adni.loni.usc.edu). The ADNI was launched in 2003 as a public-private partnership led by Principal Investigator Michael W. Weiner, MD. The primary goal of ADNI has been to test whether serial magnetic resonance imaging (MRI), positron emission tomography (PET), other biological markers, and clinical and neuropsychological assessment can be combined to measure the progression of mild cognitive impairment (MCI) and early Alzheimer's disease (AD).

^2^The code for the model and the explanation method can be found here: https://github.com/suletekkesinoglu/GCN_XAI_ADNI. The data can be obtained from the corresponding author upon reasonable request, as long as the data transfer complies with legal data protection requirements and data use agreements.

^3^Link to survey: https://forms.gle/rreCnBCgEeiqQeaP7.

## References

[B1] Ahmedt-Aristizabal D. Armin M. A. Denman S. Fookes C. Petersson L. (2021). Graph-based deep learning for medical diagnosis and analysis: past, present and future. Sensors 21, 4758. 10.3390/s21144758 34300498 PMC8309939

[B2] Anirudh R. Thiagarajan J. J. (2019). “Bootstrapping graph convolutional neural networks for autism spectrum disorder classification,” in ICASSP 2019-2019 IEEE International Conference on Acoustics, Speech and Signal Processing (ICASSP). Brighton: IEEE, 3197–3201.

[B3] Baldassarre F. Azizpour H. (2019). Explainability techniques for graph convolutional networks. arXiv.

[B4] Bogdanovic B. Eftimov T. Simjanoska M. (2022). In-depth insights into Alzheimer's disease by using explainable machine learning approach. Sci. Rep. 12, 1–26. 10.1038/s41598-022-10202-2 35444165 PMC9021280

[B5] Boyle G. J. Saklofske D. H. Matthews G. (2012). Psychological Assessment: Four volume Set. New York: SAGE Publications Ltd.

[B6] Chattopadhay A. Sarkar A. Howlader P. Balasubramanian V. N. (2018). “Grad-cam++: Generalized gradient-based visual explanations for deep convolutional networks,” in 2018 IEEE Winter Conference on Applications of Computer Vision (WACV). Lake Tahoe: IEEE, 839–847.

[B7] Danso S. O. Zeng Z. Muniz-Terrera G. Ritchie C. W. (2021). Developing an explainable machine learning-based personalised dementia risk prediction model: a transfer learning approach with ensemble learning algorithms. Front. Big Data 4, 21. 10.3389/fdata.2021.613047 34124650 PMC8187875

[B8] Defrancesco M. Marksteiner J. Kemmler G. Fleischhacker W. W. Blasko I. Deisenhammer E. A. (2017). Severity of depression impacts imminent conversion from mild cognitive impairment to Alzheimer's disease. J. Alzheimer's Dis. 59, 1439–1448. 10.3233/JAD-161135 28731429

[B9] Desikan R. S. Fan C. C. Wang Y. Schork A. J. Cabral H. J. Cupples L. A.. (2017). Genetic assessment of age-associated Alzheimer disease risk: Development and validation of a polygenic hazard score. PLoS Med. 14, e1002258. 10.1371/journal.pmed.1002289 28323831 PMC5360219

[B10] Doshi-Velez F. Kim B. (2017). Towards a rigorous science of interpretable machine learning. arXiv.

[B11] El-Sappagh S. Alonso J. M. Islam S. Sultan A. M. Kwak K. S. (2021). A multilayer multimodal detection and prediction model based on explainable artificial intelligence for Alzheimer's disease. Sci. Rep. 11, 1–26. 10.1038/s41598-021-82098-3 33514817 PMC7846613

[B12] Fischl B. (2012). Freesurfer. Neuroimage 62, 774–781. 10.1016/j.neuroimage.2012.01.021 22248573 PMC3685476

[B13] Ghanvatkar S. Rajan V. (2022). Towards a Theory-Based Evaluation of Explainable Predictions in Healthcare.

[B14] Giorgio J. Landau S. M. Jagust W. J. Tino P. Kourtzi Z. Initiative A. D. N.. (2020). Modelling prognostic trajectories of cognitive decline due to Alzheimer's disease. NeuroImage: Clini. 26, 102199. 10.1016/j.nicl.2020.102199 32106025 PMC7044529

[B15] Hill C. (2022). How the Symptoms of Alzheimer's are Related to the Brain Lobe affected.

[B16] Holzinger A. Malle B. Saranti A. Pfeifer B. (2021). Towards multi-modal causability with graph neural networks enabling information fusion for explainable AI. Inform. Fusion 71, 28–37. 10.1016/j.inffus.2021.01.008

[B17] Ilias L. Askounis D. (2022). Explainable identification of dementia from transcripts using transformer networks. IEEE J. Biomed. Health Inform. 26, 4153–4164. 10.1109/JBHI.2022.3172479 35511841

[B18] Jack C. R. Bernstein M. A. Fox N. C. Thompson P. Alexander G. Harvey D.. (2008). The Alzheimer's disease neuroimaging initiative (ADNI): MRI methods. J.Magnet. Reson. Imag. 27, 685–691. 10.1002/jmri.21049 18302232 PMC2544629

[B19] Kamal M. S. Northcote A. Chowdhury L. Dey N. Crespo R. G. Herrera-Viedma E. (2021). Alzheimer's patient analysis using image and gene expression data and explainable-AI to present associated genes. IEEE Trans. Instrum. Meas. 70, 1–7. 10.1109/TIM.2021.310705633776080

[B20] Kipf T. N. Welling M. (2016). Semi-supervised classification with graph convolutional networks. arXiv preprint arXiv:1609.02907.

[B21] Lei B. Zhu Y. Yu S. Hu H. Xu Y. Yue G.. (2023). Multi-scale enhanced graph convolutional network for mild cognitive impairment detection. Pattern Recognit. 134, 109106. 10.1016/j.patcog.2022.109106

[B22] Lombardi A. Diacono D. Amoroso N. Biecek P. Monaco A. Bellantuono L.. (2022). A robust framework to investigate the reliability and stability of explainable artificial intelligence markers of mild cognitive impairment and Alzheimer's disease. Brain Inform. 9, 1–17. 10.1186/s40708-022-00165-5 35882684 PMC9325942

[B23] Lundberg S. M. Erion G. Chen H. DeGrave A. Prutkin J. M. Nair B.. (2020). From local explanations to global understanding with explainable AI for trees. Nat. Mach. Intellig. 2, 56–67. 10.1038/s42256-019-0138-9 32607472 PMC7326367

[B24] Lundberg S. M. Lee S.-I. (2017). A unified approach to interpreting model predictions. Adv. Neural Inf. Process. Syst. 30, 4768–4777.

[B25] Mulyadi A. W. Jung W. Oh K. Yoon J. S. Suk H.-I. (2022). Xadlime: explainable Alzheimer's disease likelihood map estimation via clinically-guided prototype learning. arXiv. 10.1016/j.neuroimage.2023.120073 37037063

[B26] Parisot S. Ktena S. I. Ferrante E. Lee M. Guerrero R. Glocker B.. (2018). Disease prediction using graph convolutional networks: application to autism spectrum disorder and Alzheimer's disease. Med. Image Anal. 48, 117–130. 10.1016/j.media.2018.06.001 29890408

[B27] Parisot S. Ktena S. I. Ferrante E. Lee M. Moreno R. G. Glocker B.. (2017). “Spectral graph convolutions for population-based disease prediction,” in International Conference on Medical Image Computing and Computer-Assisted Intervention. Cham: Springer, 177–185.

[B28] Podcasy J. L. Epperson C. N. (2022). Considering sex and gender in Alzheimer's disease and other dementias. Dialogues in Clinical Neuroscience. 18, 2707–2724. 10.1002/alz.12662 PMC5286729 28179815

[B29] Pope P. E. Kolouri S. Rostami M. Martin C. E. Hoffmann H. (2019). “Explainability methods for graph convolutional neural networks,” in Proceedings of the IEEE/CVF Conference on Computer Vision and Pattern Recognition. Long Beach: IEEE, 10772–10781

[B30] Rahim N. El-Sappagh S. Ali S. Muhammad K. Del Ser J. Abuhmed T. (2022). Prediction of Alzheimer's progression based on multimodal deep-learning-based fusion and visual explainability of time-series data. Inform. Fusion. 92, 363–388. 10.1016/j.inffus.2022.11.028

[B31] Raji C. A. Lopez O. Kuller L. Carmichael O. Becker J. (2009). Age, Alzheimer disease, and brain structure. Neurology 73, 1899–1905. 10.1212/WNL.0b013e3181c3f293 19846828 PMC2788799

[B32] Rakhimberdina Z. Liu X. Murata T. (2020). Population graph-based multi-model ensemble method for diagnosing autism spectrum disorder. Sensors 20, 6001. 10.3390/s20216001 33105909 PMC7660214

[B33] Rakhimberdina Z. Murata T. (2019). “Linear graph convolutional model for diagnosing brain disorders,” in International Conference on Complex Networks and Their Applications. Cham: Springer, 815–826.

[B34] Reuter M. Schmansky N. J. Rosas H. D. Fischl B. (2012). Within-subject template estimation for unbiased longitudinal image analysis. Neuroimage 61, 1402–1418. 10.1016/j.neuroimage.2012.02.084 22430496 PMC3389460

[B35] Robnik-Šikonja M. Kononenko I. (2008). Explaining classifications for individual instances. IEEE Trans. Knowl. Data Eng. 20, 589–600. 10.1109/TKDE.2007.190734

[B36] Schwarzenberg R. Hübner M. Harbecke D. Alt C. Hennig L. (2019). Layerwise relevance visualization in convolutional text graph classifiers. arXiv. 10.18653/v1/D19-5308

[B37] Velazquez M. Lee Y. Initiative A. D. N. (2021). Random forest model for feature-based Alzheimer's disease conversion prediction from early mild cognitive impairment subjects. PLoS ONE 16, e0244773. 10.1371/journal.pone.0244773 33914757 PMC8084194

[B38] Vilone G. Longo L. (2020). Explainable artificial intelligence: a systematic review. arXiv.

[B39] Vina J. Lloret A. (2010). Why women have more Alzheimer's disease than men: gender and mitochondrial toxicity of amyloid-β peptide. J. Alzheimer's Dis. 20, S527–S533. 10.3233/JAD-2010-100501 20442496

[B40] Wu F. Souza A. Zhang T. Fifty C. Yu T. Weinberger K. (2019). “Simplifying graph convolutional networks,” in International Conference on Machine Learning. New York: PMLR, 6861–6871.

[B41] Yao D. Liu M. Wang M. Lian C. Wei J. Sun L.. (2019). “Triplet graph convolutional network for multi-scale analysis of functional connectivity using functional MRI,” in International Workshop on Graph Learning in Medical Imaging. Cham: Springer, 70–78.

[B42] Ying Z. Bourgeois D. You J. Zitnik M. Leskovec J. (2019). Gnnexplainer: Generating explanations for graph neural networks. Adv. Neural Inf. Process. Syst. 32, 9244–9255. 32265580 PMC7138248

[B43] Zhang Y. Weng Y. Lund J. (2022). Applications of explainable artificial intelligence in diagnosis and surgery. Diagnostics 12, 237. 10.3390/diagnostics12020237 35204328 PMC8870992

[B44] Zhou H. He L. Zhang Y. Shen L. Chen B. (2022). “Interpretable graph convolutional network of multi-modality brain imaging for Alzheimer's disease diagnosis,” in 2022 IEEE 19th International Symposium on Biomedical Imaging (ISBI). Kolkata: IEEE. 10.1109/ISBI52829.2022.9761449

