# Supplementary material for: Explaining graph convolutional network predictions for clinicians—An explainable AI approach to Alzheimer's disease classification
Source: Front Artif Intell. 2024 Jan 8;6:1334613. doi: 10.3389/frai.2023.1334613 (PMC10801225; doi:10.3389/frai.2023.1334613)
Supplement: Supplementary file 1 [file Data_Sheet_1.PDF]

Supplementary Material

1 SUPPLEMENTARY DATA

Link to survey: `https://forms.gle/rreCnBCgEeiqQeaP7`

2 SUPPLEMENTARY TABLES AND FIGURES

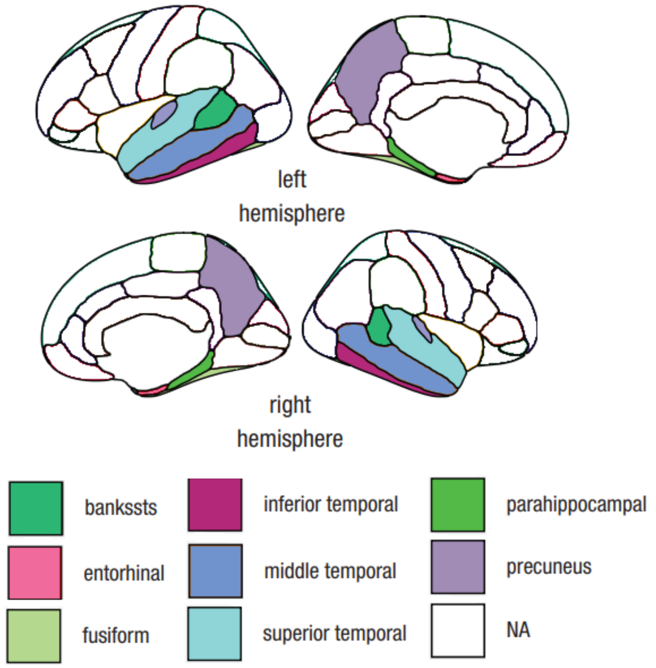

Figure S1. Selected regions shown in structural image analysis

Table S1. Influence of individual features on classification results.

Selected Features	Precision %			Recall %			F1 %			MCF	MCA
	NC	MCI	AD	NC	MCI	AD	NC	MCI	AD		
All Features	73.83	86.96	84.78	91.86	63.16	95.12	81.87	73.17	89.66	81.56	80.18
All-Age	73.83	89.06	80.39	91.86	60	100	81.86	71.7	89.13	80.89	79.72
All-Gen	73.83	89.06	80.39	91.86	60	100	81.86	71.7	89.13	80.89	79.72
All-MCT	73.83	89.06	80.39	91.86	60	100	81.86	71.7	89.13	80.89	79.72
All-THV	73.83	88.1	83.33	91.86	62.11	97.56	81.86	72.84	89.88	81.53	80.18
All-Gen, -Age, -MCT	73.83	88.71	77.36	91.86	57.89	100	81.86	70.06	87.23	79.72	78.82
All-Gen,-Age,-THV	73.83	88.89	78.85	91.86	58.95	100	81.86	70.89	88.17	80.31	79.28
All -MEM	73.83	86.96	84.78	91.86	63.16	95.12	81.87	73.17	89.66	81.56	80.18
All-EXF	74.31	92.31	85.41	94.19	63.16	100	83.08	75	92.14	83.40	81.98
All-LAN	74.55	92.31	85.11	95.35	63.16	97.56	83.67	75	90.91	83.19	81.98
All-Gen,-Age and -MEM	73.58	87.88	82	90.7	61.05	100	81	72.05	90.11	81.13	79.72
All-Gen,-Age and -EXF	74.38	87.88	80.39	90.7	61.05	100	81.67	72.05	89.13	80.95	79.72
All-Gen,-Age and -LAN	73.83	89.06	80.39	91.86	60	100	81.86	71.7	89.13	80.89	79.72
All-GDS	73.83	88.23	85.11	91.63	63.16	97.56	81.86	73.62	90.91	82.13	80.63
All-Gen,-Age and -GDS	74.29	87.88	80.39	90.7	61.05	100	81.67	72.05	89.13	80.95	79.72
All-MoCA	74.31	90.77	83.33	94.19	62.11	97.56	83.07	73.75	89.89	82.24	81.08
All-MM	74.55	92.19	83.33	95.35	62.11	97.56	83.67	74.21	89.89	82.59	81.53
All-Aβ	73.83	89.06	80.39	91.86	60	100	81.86	71.7	89.13	80.89	79.72
All-TAU	74.55	93.22	77.36	95.35	57.9	100	83.67	71.43	87.23	80.78	80.18
All-Aβ and -TAU	67.77	91.67	77.36	95.35	46.32	100	79.23	61.54	87.23	75.99	75.23
All-PHS	74.31	91.80	78.85	94.18	58.95	100	83.08	71.8	88.17	81.01	80.18

Table S2. Feature discretization by grouping continuous data into the set of bins based on the distance from the mean, measured in standard deviation (SD).

	MCT(mm)	THV(cm^3)	MEM	EXF	LAN	PHS
Very low	$2<t_k \leq 2.37$	$3.59<t_k \leq 4.73$	$-2<t_k \leq -1.15$	$-2<t_k \leq -0.763$	$-1.09<t_k \leq -0.55$	$-2.27<t_k \leq -1.61$
Low	$2.38<t_k \leq 2.56$	$4.74<t_k \leq 5.51$	$-1.14<t_k \leq -0.41$	$-0.762<t_k \leq -0.145$	$-0.56<t_k \leq -0.01$	$-1.62<t_k \leq -0.95$
Nominal	$2.57<t_k \leq 2.75$	$5.52<t_k \leq 6.26$	$-0.42<t_k \leq 0.34$	$-0.146<t_k \leq 0.481$	$-0.01<t_k \leq 0.54$	$-0.94<t_k \leq -0.29$
Not so high	$2.76<t_k \leq 2.94$	$6.27<t_k \leq 7.02$	$0.35<t_k \leq 1.08$	$0.482<t_k \leq 1.112$	$0.54<t_k \leq 1.08$	$-0.3<t_k \leq 0.37$
High	$2.95<t_k \leq 3.13$	$7.03<t_k \leq 7.78$	$1.09<t_k \leq 1.83$	$1.113<t_k \leq 2$	$1.09<t_k \leq 1.62$	$0.38<t_k \leq 1.03$
Very high	$3.14<t_k \leq 3.3$	$7.79<t_k \leq 8$	$1.84<t_k \leq 2.58$	$1.732<t_k \leq 2.4$	$1.63<t_k \leq 2.17$	$1.04<t_k \leq 1.69$

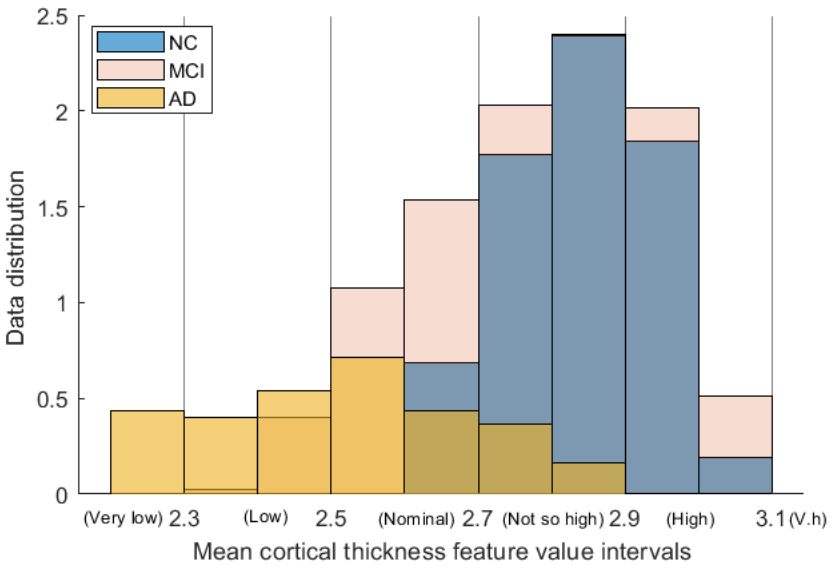

Figure S2. Feature discretization for Mean Cortical Thickness

Table S3. Confusion matrix on the domain expert's prediction.

	NC	MCI	AD
NC	10	1	-
MCI	2	11	9
AD	-	-	22

The rows correspond to the true class, and the columns correspond to the predicted class.

Table S4. Confusion matrix on the domain expert's presumption of the AI model's prediction

	NC	MCI	AD
NC	6	5	-
MCI	13	9	-
AD	-	2	20

The rows correspond to the true class, and the columns correspond to the predicted class.

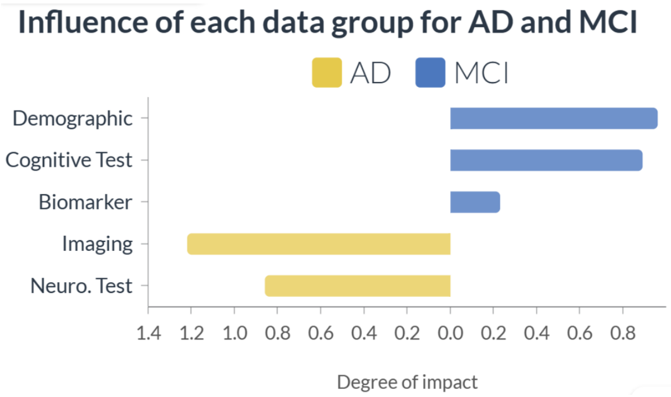

Figure S3. An alternative visualization for the influence of each data group on the factual and counterfactual classes
